# A meta-analysis of the effects of ketamine on suicidal ideation in depression patients

**DOI:** 10.1038/s41398-024-02973-1

**Published:** 2024-06-10

**Authors:** ZuoYao Shen, DaiQuan Gao, Xue Lv, HongXing Wang, WeiHua Yue

**Affiliations:** 1 The First Affiliated Hospital of Xinxiang Medical College Henan, Henan, China; 2 Peking University Sixth Hospital, Peking University Institute of Mental Health, Beijing, China; 3 NHC Key Laboratory of Mental Health (Peking University), National Clinical Research Center for Mental Disorders (Peking University Sixth Hospital), Beijing, China; 4 Research Unit of Diagnosis and Treatment of Mood Cognitive Disorder, Chinese Academy of Medical Sciences (No.2018RU006), Beijing, China; 5 Department of Neurology, Xuanwu Hospital, Capital Medical University, Beijing, China

**Keywords:** Clinical pharmacology, Depression

## Abstract

The treatment of suicidal ideation in patients with depression has been a major problem faced by psychiatric and emergency departments, and reasonable drug selection is particularly important. Ketamine has been shown to reduce suicidal ideation rapidly, but the strength of the effect is unclear and there is little evidence-based medical evidence to support this. We systematically searched all articles published on PubMed, Cochrane Library, Web of Science, CNKI and EMBASE. Stata 15 and R 4.1.3 were used for meta-analysis, and odds ratios were calculated in fixed effects or random effects models based on the heterogeneity test results. Our search resulted in 505 articles; we analyzed 14 studies, which included 1,380 participants. The 14 studies included 10 randomized controlled trial (RCT) studies and 4 single-arm studies. Our study suggests that, ketamine has a significant therapeutic effect on suicidal ideation throughout the treatment cycle. We performed network meta-analyses(NMA) and pairwise meta-analyses to compare the efficacy of ketamine in the reduction of suicidal ideation. There was a significant reduction in suicidal ideation within the first day after treatment (NMA ketamine day1 RR = 10.02, 95%CI = 4.24 to 23.68). In repeated treatment, the degree of recovery of suicidal ideation after the last dose was significantly greater than that after the first dose (RR = 0.56, 95%CI = 0.51 to 0.62). Recovery of suicidal ideation was also significantly better in the treatment end point than in the placebo group at the same time point (NMA ketamine day26 RR = 4.29, 95%CI = 1.41 to 13.08). This is the first network meta-analysis to demonstrate the role of ketamine in the alleviation of suicidal ideation. Our network meta-analysis also compared the effects of different drugs at different time points, which was not done in previous studies. This is of great reference significance for future drug research andrational drug use.

## Introduction

Suicide is a major public health problem worldwide, with more than 800,000 deaths and about 16 million attempted suicides worldwide each year, and the incidence of suicide has steadily increased over the past 20 years [1–3]. A large number of acute suicidal depression patients are admitted to the emergency room each year [4]. In the absence of specific treatment for acute suicidal ideation, suicidal patients are often hospitalized only briefly to stabilize their condition and are discharged before psychopharmacological treatment shows efficacy. And for people with suicidal depression, only a third or less achieve remission, and less than half achieve 50% remission with typical first-line drugs [5]. For suicidal patients who need to be treated quickly, even before a thorough diagnosis is established, it is important to identify a drug that can provide rapid relief and reduce suicidal ideation.

Current treatments to reduce suicidal ideation and behavior occurrence, such as antidepressants, lithium, clozapine, psychotherapy, electroconvulsive therapy, and transcranial magnetic stimulation, are numerous, but are not immediately effective for all patients. Standard antidepressants have proven effective in treating suicidal ideation, but they take a relatively slow time to work, usually four to six weeks for optimal results. In addition, selective serotonin reuptake inhibitors warn of a potential increase in suicidal ideation in children and adolescents [6].

A growing body of research suggests that ketamine and esketamine may rapidly reduce suicidal ideation in depressed subjects at risk for suicide [7–11]. There have also been studies showing that even the most resistant patients can benefit from ketamine, and that medium to long-term treatment is effective for many patients [12].

The purpose of our study was to determine whether ketamine can rapidly improve the reduction of suicidal ideation behavior in patients with depression in the short term, and to explore the change in the intensity of its effect over time.

## Methods

We performed network meta-analyses(NMA) and pairwise meta-analyses to compare the efficacy of ketamine in the reduction of suicidal ideation. NMA compares the efficacy of drugs at different time points. The effect intensity between the two can be directly compared, and the probability ranking prediction analysis and comparison of the strength of the therapeutic effect. A pairwise meta-analysis was performed to compare the effect of the first and last treatment in the repeated dosing group. The difference between the first and the last treatment was used to determine whether repeated dosing could bring greater benefits to ketamine treatment.

### Search strategy

The retrieval strategy takes ketamine, suicide and depression as search terms, and searches their subject words and all entry terms. Combined with subject terms and entry terms, a comprehensive literature search was conducted in Cochrane Library, Embase, Web of science, CNKI, PubMed to retrieve all studies published in the last ten years up to August 1, 2022.

### Study selection

Outpatients and inpatients aged between 18 and 70 years with depression as their primary psychiatric diagnosis. The included studies can be RCT studies or single-arm studies, which should meet the requirement that the enrolled patients have been assessed by strict relevant scales and have definite suicidal ideation (C-SSRS, CGI-SS-r, BSS, MADRS-SI, SSI part I). Studies with no baseline suicidal ideation rating data were excluded. Excluded were studies in which multiple therapies were used in combination with therapy (e.g., Physical therapy combined with drug therapy) and in which medication was changed during the study, as well as studies in which patients were enrolled with primary psychotic disorders (e.g., schizophrenia, schizoaffective disorder, etc.), dissociative disorders, pervasive developmental disorders, cognitive disorders, or anorexia nervosa.

We included different ketamine-related studies, including all elastic dose studies, which allow researchers to titrate to an adequate dose appropriate for individual patients. For fixed-dose studies, we included the target dose to the maximum dose based on the international consensus study on antipsychotic dosing [13]. Due to the limited number of included studies, for studies with single and repeated ketamine administration, we analyzed them together.

### Network meta-analysis

Unlike paired meta-analyses, which allow one intervention to be compared with another on the basis of head-to-head data from randomized trials, NMA facilitate simultaneous comparisons of the efficacy or safety of multiple interventions that may not be directly compared in randomized trials [14, 15].

### Quality assessment

Our analysis included all published RCTs and single-arm studies. For RCTs, we applied the Cochrane Risk of Bias tool 2 (RoB 2) to assess the risk of bias [16]. For single-arm studies, we applied the Methodological Index for Non-Randomized Studies (MINORS) score to assess the risk of bias [17]. Study quality was assessed independently by two investigators (ZS and DG), strictly following Cochrane RoB 2 and MINORS bias risk assessment method. If there are disagreements, resolve them through discussion.

### Data extraction

The primary outcome measure of our study was the level of remission of suicidal ideation after medication in depressed patients with definite suicidal ideation. Therefore, we extracted all relevant binary and continuous outcome data from baseline to endpoint. However, due to the inconsistencies of the suicidal ideation assessment scales used in most studies, and relatively few studies meeting the criteria included, we have to give up the processing of the continuous outcome data. By redefining whether patients achieve effective remission of suicidal ideation, the results of binary data under different scales are unified (that is, the level of suicidal ideation of patients is reduced to (SSI) part I score < 2, QIDS-SR16 < 1,CGI-SS: 0 or 1, MADRS-SI ≤ 2 or SSI total score ≤ 3 were considered as effective mitigation, and the binary outcome data were extracted by judging whether effective mitigation was achieved or not). All the data is done and screened independently by the two authors (ZY S and DQ G), who cannot interfere with each other’s decisions. When there is disagreement between two authors, the third author (XL) clarifies and makes the final decision.

### Data processing

Data transformation is carried out for studies containing data of multiple time nodes. For example, if a study has relevant data for a treatment at multiple time points, we will treat the data at each time point as a unique intervention. The previous study was converted into a multi-arm study on which we conducted the NMA (Supplementary Fig. 1).

### Data analysis

The data were analyzed from 20 August 2022 to 10 September 2022. We performed pairwise meta-analysis and NMA in a Bayesian environment using Stata 15 and R 4.1.3 software. For pairwise meta-analysis and NMA, we flexibly selected fixed effects model and random effects model according to the heterogeneity between studies, and determined the heterogeneity of meta-analysis by *I*^*2*^ (threshold 50%). We extracted binary results in our analysis and calculated the effect size by relative ratio (RR) of the number of people with effective mitigation from different studies. The NMA synthesizes direct and indirect evidence of relevant therapeutic effects across all time nodes to provide an estimate of maximum efficacy. We compared the effects of different strategies using Surface under the Cumulative Ranking Curve (SUCRA), allowing for a comparative analysis of the intensity of drug action at each time node based on the surface under the cumulative ranking. SUCRA values range from 0 to 100%. The higher the SUCRA value, and the closer to 100%, the higher the likelihood that a therapy is in the top rank or one of the top ranks; the closer to 0 the SUCRA value, the more likely that a therapy is in the bottom rank, or one of the bottom ranks [15, 18]. The inconsistencies between direct evidence and indirect evidence were calculated by node splitting method. Publication bias was evaluated by funnel plot and Egger’s test. All statistical differences were considered significant when the *p* < *0.05*.Subgroup analyses were performed to explore heterogeneity.

## Results

### Descriptions of the included studies

**Fig. 1:**
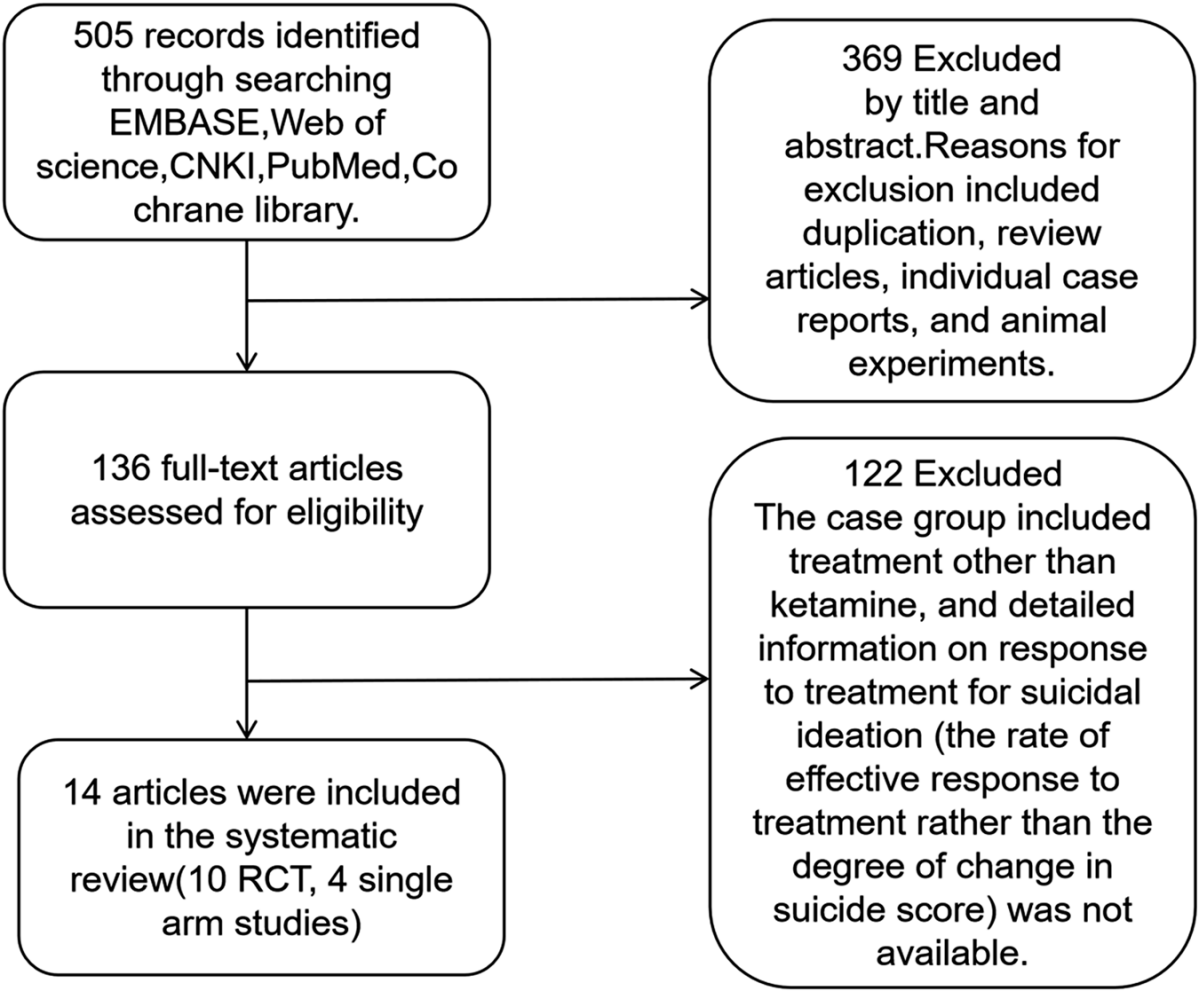
PRISMA diagram. Flow diagram showing the study selection process.

A total of 505 studies published before August 2022 were retrieved. After reading titles and abstracts, 369 studies were excluded. Reasons for included duplication, review articles, individual case reports, and animal experiments. 136 studies were included in the full text analysis, and 14 studies meeting the requirements were finally identified (Table 1). There were 1380 participants in 14 studies. Among them, 10 were RCT studies and 4 were single-arm studies. The mode of administration in these studies included both single and repeated doses. We conducted a pairwise meta-analysis of first-to-last treatment in six studies of repeated dosing to determine whether it is superior to a single dose. The PRISMA flowchart is shown in Fig. 1. The risk assessment of bias for RCTs and non-RCTs is shown in supplementary materials Tables 1 and 2.

**Table 1:** Included studies list.

Study	Year	Race	Method	Frequency	Primary intervention	Blind assessment	Type of patients	Type of treatment setting	Type of effect	Sample size	Age (years) Mean/median (range)	Male (%)	Trial duration	Diagnostic tools	Suicidal ideation remission definition	Ketamine dosage
Zheng et al. [43]	2022	Asian	Intravenous	Repeatedly	Ketamine	Single arm	MDD or BD	Inpatient	Long-term duration	60	35.3 (12.4)	27 (45%)	12 day	DSM-V	SSI part I < 2	0.5 mg/kg
Abbar et al. [44]	2021	European	Intravenous	Repeatedly	Ketamine/Placebo	Double-Blinded	Bipolar, depressive	Inpatient	Acute	73/83	38 (18–75)/41 (18–76)	19 (26.0)/31 (37.3)	6 weeks	DSM-IV	SSI score of 0 to 3	0.5 mg/kg
Feeney et al. [45]	2021	American	Intravenous	Single	Ketamine/midazolam	Double-Blinded	MDD	Outpatients	Long-term duration	40/16	45.75 ± 12.32	28 (50%)	3 day	DSMIV-TR TM	MADRS suicide item< 2	0.1 mg/kg, 0.5 mg/kg, 1.0 mg/kg
Domany et al. [46]	2021	American	Intranasal	Single	Ketamine/Placebo	Double-Blinded	Mental disorders	Inpatient	Acute	15/15	35.11 (8.67)/35.78 (9.86)	4 (44.4%) /4 (44.4%)	4 h	NR	MADRS suicide item=0	40 mg
Canuso et al. [47]	2021	American	Intranasal	Repeatedly	Esketamine/Placebo	Double-Blinded	MDD	Inpatient	Acute	222/224	40.5 (12.92) /39.6 (13.08)	92 (40.7) /85 (37.8)	4 weeks	DSM-V	CGI-SS-r score of 0 to 1	84 mg
Zhou et al. [48]	2020	Asian	Intravenous	Repeatedly	Ketamine	Single arm	Depression	Inpatient	Long-term duration	73	35.2(11.8)	35(47.9)	12 day	DSM-V	SSI part I < 2	0.5 mg/kg
Canuso et al. [49]	2018	American	Intranasal	Repeatedly	Esketamine/Placebo	Double-Blinded	Major depression	Inpatient	Acute	35/31	35.7 (13.40) /36.0 (12.82)	13 (37.1) /10 (32.3)	4 weeks	DSM-IV-TR	CGI-SS-r score of 0 to 1	84 mg
McIntyre et al. [7]	2020	American	Intravenous	Repeatedly	Ketamine	Single arm	MDD or BD	Outpatients	Long-term duration	213	45 (15)	95 (44.6)	2 weeks	DSM-V	QIDS-SR16 = 0	0.5 ~ 0.75 mg/kg
Price et al. [50]	2014	American	Intravenous	Single	Ketamine/Midazolam	Double-Blinded	TRD	Outpatients	Acute	36/21	48.6 (11.4)/43.8 (10.9)	16 (44%)/11 (52%)	24 h	DSM-IV-TR	QIDS-SR16 = 0	0.5 mg/kg
Ionescu et al. [51]	2019	American	Intravenous	Repeatedly	Ketamine/Placebo	Double-Blinded	MDD	Outpatients	Long-term duration	13/13	45.5 (13.6) /45.3 (11.7)	6 (46%) /10 (77%)	3 weeks	DSM-IV	C-SSRS SI score = 0	0.5 mg/kg
Domany et al. [52]	2019	American	Intravenous	Single	Ketamine/Placebo	Double-Blinded	MDD, BD, depression, dysthymia	Inpatient	Acute	9/9	35.11 (8.67)/35.78 (9.86)	4 (44.4%)/4 (44.4%)	24 h	MINI	MADRS suicide item score of 0 to 2	0.2 mg/kg
Grunebaum et al. [9]	2018	American	Intravenous	Single	Ketamine/midazolam	Double-Blinded	MDD	Inpatient	Acute	40/40	38.4 (13.2)/40.7 (13.1)	18(45%)/14 (35%)	24 h	DSM-IV	SSI <3	0.5 mg/kg
Burger et al. [53]	2016	American	Intravenous	Single	Ketamine/Placebo	Double-Blinded	Depression	Inpatient	Acute	3/7	28/27	2 (67%)/5 (71%)	4 h	NR	SSI score of 0 to 3	0.2 mg/kg
Yanni et al. [54]	2019	Asian	Intravenous	Repeatedly	Ketamine	Single arm	Depression	Inpatient	Long-term duration	86	33.99 (11.65)	43 (50.0%)	12 day	DSM-V (SCID-5)	SSI part I < 2	0.5 mg/kg

*DSM-IV* Diagnostic and Statistical Manual of Mental Disorders, Fourth Edition, *DSM-IV-TR* Diagnostic and Statistical Manual of Mental Disorders, Fourth Revised Edition, *DSM-V* Diagnostic and Statistical Manual of Mental Disorders, Fifth Edition, *MINI* Concise International Interview Examination for Neuropsychiatric disorders, *SSI* Baker Scale of Suicidal Ideation, *C-SSRS*, Columbia Severe Suicide Scale, *MADRS suicide item* Montgomery Scale suicidal ideation item, *QIDS-SR16-si* Self-report of depressive symptoms 16 quick list suicide items, *CGI-SS-r* Clinical global impression - Suicide Severity - revised, *NR* not reported

### Network meta-analysis

**Fig. 2:**
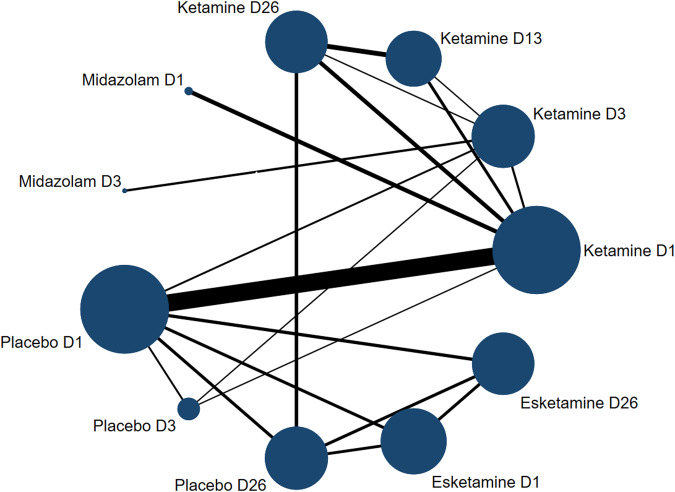
Overall efficacy network evidence figure. The size of the node corresponds to the number of participants assigned to each treatment. Treatments with direct comparisons are connected by a line; Its thickness corresponds to the number of tests compared. D1: Day 1, D3: day 3, D13: day 13, D26: day 26.

We conducted a comprehensive comparison of the alleviating effect of suicidal ideation at different time points after the administration of different treatment strategies, obtained the direct and indirect comparison results between different treatment measures at each time point through the method of NMA, and conducted a comprehensive ranking comparison of the remission degree at different time points. A total of 14 studies with 1380 participants were included. The overall characteristics of the included studies are shown in Fig. 2.

**Fig. 3:**
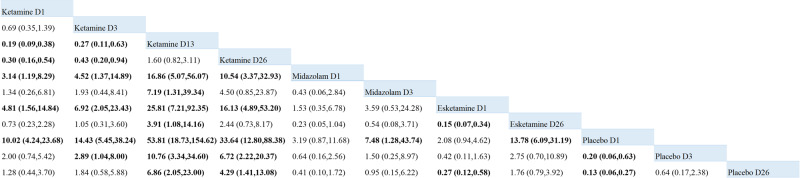
Network meta-analysis comparing the remission level of suicidal ideation with all treatment measures at different time points. Important results are shown in bold. D1: Day 1, D3: day 3, D13: day 13, D26: day 26.

**Fig. 4:**
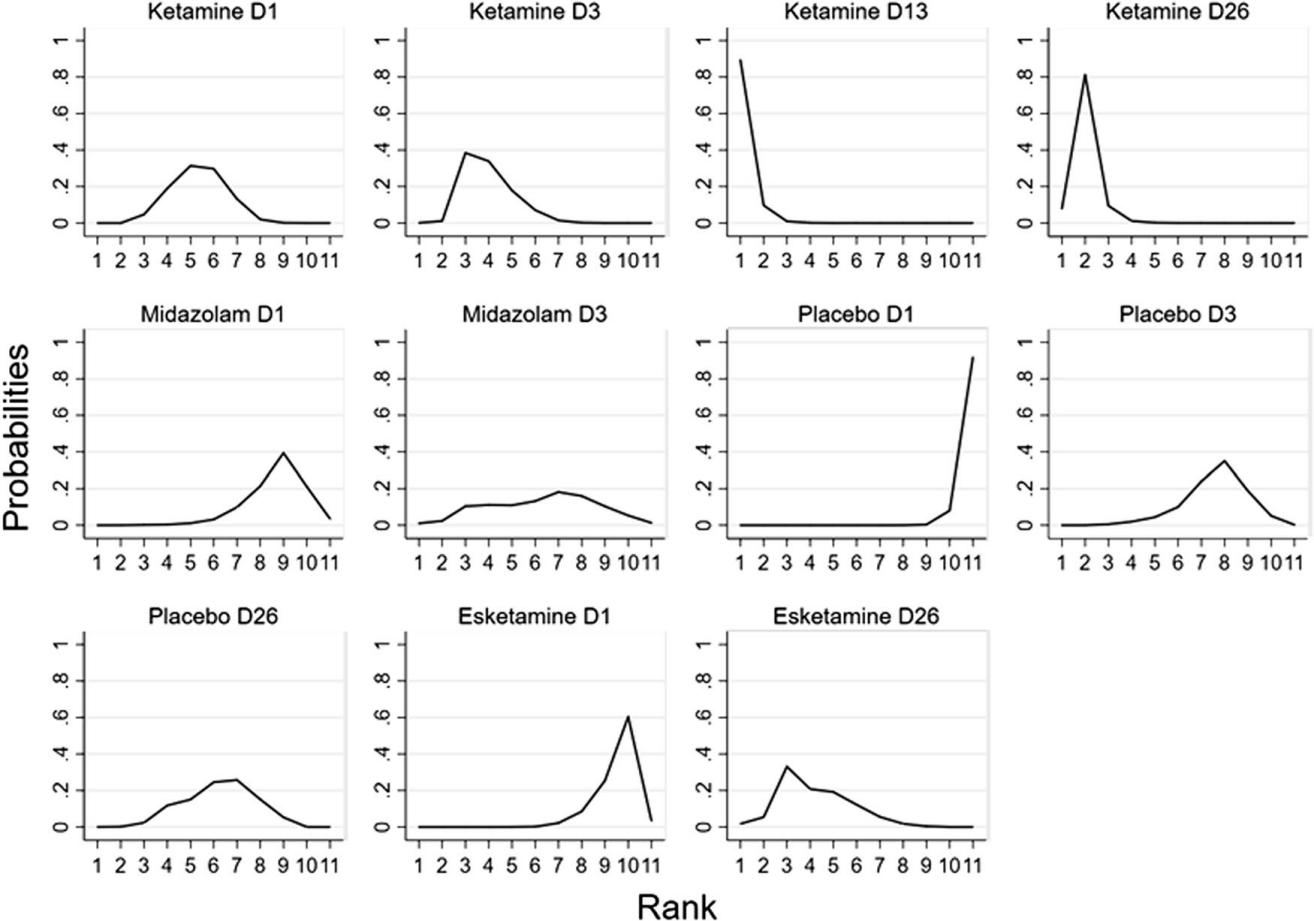
Probability ranking of the effects of different treatment measures on the remission level of suicidal ideation under multiple time nodes.

After the first day of treatment, ketamine was significantly more effective than placebo (RR = 10.02, 95%CI = 4.24–23.68), esketamine (RR = 4.81, 95%CI = 1.56–14.84), and midazolam (RR = 3.14, 95%CI = 1.19–8.29). In addition, ketamine was significantly better than placebo at all the same time points. Ketamine D3 (RR = 2.89, 95%CI = 1.04–8.00), Ketamine D26 (RR = 4.29, 95%CI = 1.41–13.08). In the placebo group, the results on day 3 (RR = 0.20, 95%CI = 0.06–0.63) and day 26 (RR = 0.13, 95%CI = 0.06 to 0.27) were also significantly different from those on day 1. The effects of esketamine and midazolam were not significant at the same time points compared with placebo (Fig. 3). The probability ranking of the effects of different treatment measures on the remission level of suicidal ideation at multiple time nodes is shown in Fig. 4. Cumulative probability of different treatment measures affecting the remission level of suicidal ideation under multiple time nodes is shown in Supplementary Fig. 2. The pairwise comparison contributions of NMA and the risk of bias plots are shown in Supplementary Figs. 4 and 5.

By estimating SUCRA values, we ranked the remission degree of suicidal ideation for all treatment measures at different time points. The higher the SUCRA value, the greater the possibility of suicidality ideation remission, indicating that the treatment plan at this time node has a higher degree of impact on suicidality ideation remission.

For the remission of suicidal ideation in depressed patients, the 11 groups were ranked from highest to lowest as follows: KetamineD13 (99.1%), KetamineD26 (89.6%), EsketamineD26 (70.6%), KetamineD3 (70.0%), KetamineD1 (54.8%), PlaceboD26 (50.5%), MidazolamD3 (46.2%), PlaceboD3 (32.4%), MidazolamD1 (22.1%), EsketamineD1 (14.2%), PlaceboD1 (0.4%) (Supplementary Table 3).

### Pairwise meta

**Fig. 5:**
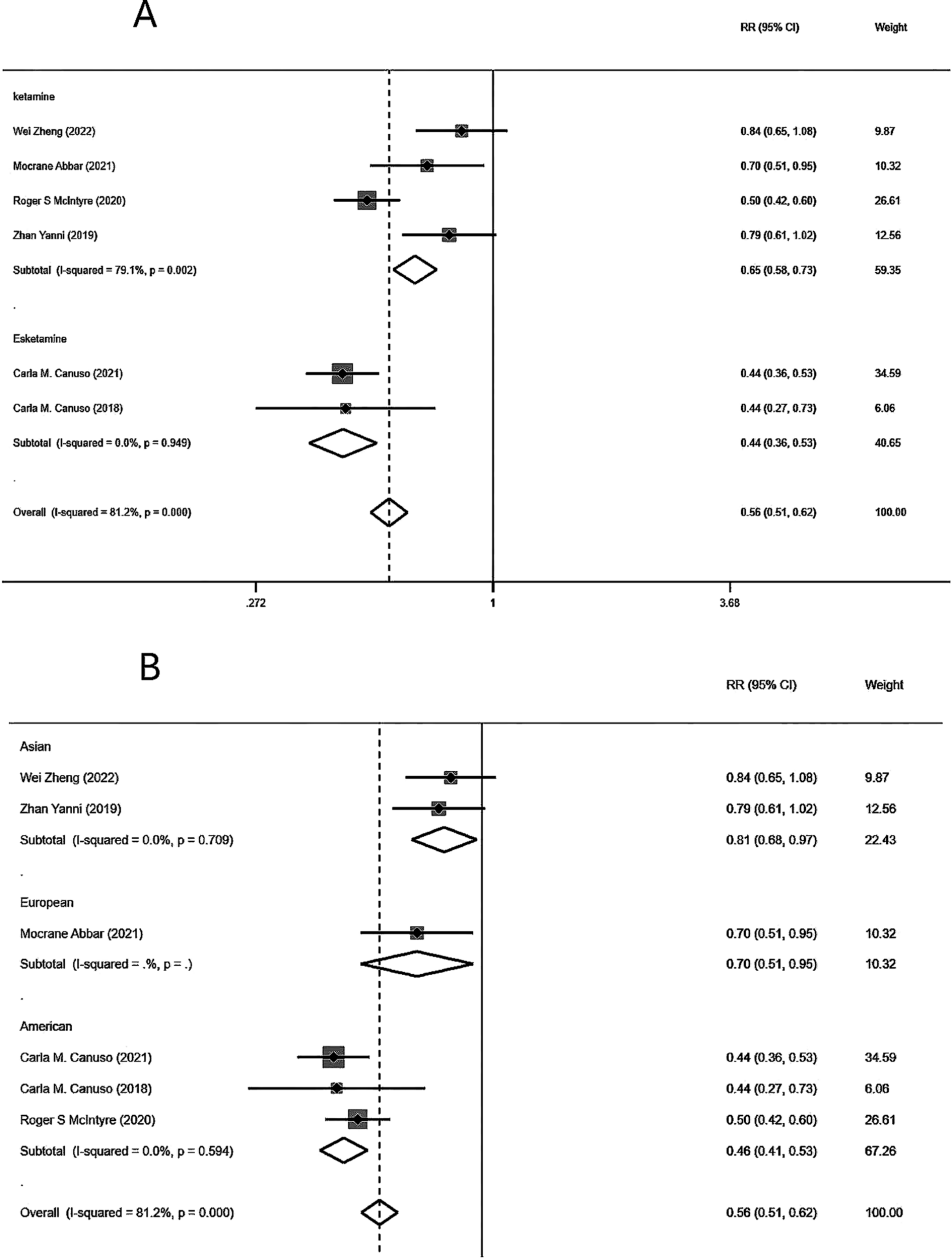
Comparison of suicidal ideation between the first and last day of ketamine treatment with repeated dosing. **A** Meta-analysis of the remission effect of suicidal ideation between the first and the last treatment in the repeated administration group. **B** Meta-analysis of the effect of suicidal ideation remission after the first and last treatment in different race.

In the repeated administration group (including ketamine and esketamine), there was a significant difference in the remission of suicidal ideation between the first day of treatment and the last treatment (RR = 0.56, 95%CI = 0.51–0.62). The overall results were heterogeneous (*I*^2^ = 81.2%, *P* = 0.000) and this heterogeneity persisted in the ketamine versus esketamine subgroups (*I*^2^ = 79.1%, *P* = 0.002). We then performed subgroup analyses according to race, and there was no longer heterogeneity (Fig. 5A, B). Egger’s publication bias test and the funnel plot are shown in Supplementary Figs. 5 and 6.

## Discussion

Although previous reviews and meta-analyses have provided valuable insights into the effects of specific medications on suicidal behavior in specific populations, none have been able to address the strength of early drug action in depressed suicidal patients [19–23]. Ketamine is a promising treatment option for patients with depression and rapid reduction of suicidal ideation in patients. The acute efficacy of ketamine for depression has been demonstrated in repeated RCTS [24, 25]. However, there has been very limited generalization of relevant RCT data in real practice. This has brought great resistance for many researchers to carry out relevant evidence-based medicine verification in this respect [12]. Therefore, we further processed the data to include data from multiple follow-up points in a single study to construct the model and NMA data at multiple time levels, which expanded our network model and made our results more robust.

Our study confirms the role of ketamine in the alleviation of suicidal ideation in depressed patients. However, due to the relatively small number of included studies, we included studies that included both outpatients and inpatients with depression, MDD and TRD, single and repeated ketamine doses for analysis. It is mainly aimed at depression patients with suicidal ideation. This needs further research and analysis in the future to distinguish the specific efficacy for different patient populations.

To our knowledge, this is the first NMA to simultaneously compare the effects of different treatment strategies at different time points. In addition, our pairwise meta-analysis of the first versus the last treatment difference results highlighted the additional benefit of repeated dosing in terms of resolution of suicidal ideation. This provides strong evidence for the choice of medication for acute suicidal ideation in patients with depression.

By using Stata software, we calculated and ranked the probability of alleviating suicidal ideation in different time nodes, as shown in Fig. 4. Through the observation and comparison of the relief efficacy at different time points in the placebo group, we understand the approximate recovery progress of the natural remission of suicidal ideation in depressed patients over time. To provide a rough assessment of the outcome by looking at the overall outcome of the probability ranking, ketamine treatment for depressive patients with severe suicidal ideation showed a higher degree of recovery on day 1 of suicidal ideation than on day 3 of placebo and a similar level as on day 26 of placebo. When the placebo group was used to assess remission after ketamine treatment as a natural remission level, the improvement in suicidal ideation after day 1 of ketamine treatment was similar to the level of natural recovery at about 26 days in non-users. However, this is not a rigorous calculation, and although SUCRA results showed such a ranking strength, there was no significant difference in NMA results between ketamine on day 1 and placebo on day 3 and 26. Therefore, we can only indirectly guess the strength of ketamine in this way, and the true strength of its effect needs to be confirmed by further research.

There are also interesting findings from our study. The reduction in suicidal ideation in the placebo group was significantly different on day 3 than on day 1. This represents a presumably natural process of recovery in depressed patients with suicidal ideation. This suggests that intervention in the acute phase of patients with suicidal ideation is necessary. The way of intervention is not limited to drugs, as long as patients can be reasonably and effectively controlled in the early stage, a considerable number of patients will achieve effective remission over time.

The difference in the intensity of ketamine and esketamine in alleviating suicidal ideation may be caused to some extent by different dosage forms. In the included studies, ketamine was mainly administered intravenously, with the dosage ranging from 0.1 to 1.0 mg/kg, and in most studies, 0.5 mg/kg. One randomized controlled trial showed that the efficacy of higher doses in antidepressants was similar to that of standard doses and was well tolerated [26]. Esketamine was mainly administered intranasally at a fixed dose of 84 mg. Studies have shown that the maximum plasma concentration can be reached within 1 min after intravenous injection [27]. When the antidepressant dose was given for more than 40 min, the concentration gradually decreased after the infusion ended [28]. Intramuscular administration peaks within 5–30 min, sublingual administration peaks within 45 min, and oral administration peaks within 20–120 min. Ketamine bioavailability varied widely among non-intravenous formulations, ranging from approximately 90% intramuscular, 50% intranasal, 29% sublingual, and 17% oral [29–31].

In terms of antidepressants, a number of meta-analyses in recent years have consistently concluded that a single intravenous administration of ketamine has a rapid antidepressant effect, reaching a peak within 24 h and lasting 3 to 7 days [32–34]. Repeated infusions achieve higher remission rates and extend the time to relapse of depression [35–37]. The results of our pairwise meta-analysis, at the same time, also demonstrate the additional benefit of repeated dosing in the remission of suicidal ideation. This underscores the importance of repeated dosing in the clinical setting for depressed patients with suicidal ideation. However, due to the limited number of included studies, we could not compare the differences in drug efficacy between different dosage forms. Considering drug utilization and pharmacokinetics, it is possible that this difference in drug administration has a similar effect on suicidal ideation and depression, and further research is needed in the future.

The NMA results showed that the optimal therapeutic effect was presented on the 13th day. This was only a rough calculation and prediction of the recovery of depressed patients after ketamine treatment based on the current limited research data, without assessing the remission of suicidal ideation at other treatment points in the middle, so we could only speculate that the optimal therapeutic effect was near the 13th day.

As for the recovery degree of the 13th day is significantly better than that of the 26th day, we believe there are mainly two reasons as follows. First, pharmacokinetic factors, after a single infusion, plasma levels of ketamine were not detected for 24 h, but very low levels of ketamine metabolites were detected for 3 consecutive days and 3 consecutive days, and repeated infusion reduced ketamine clearance [38, 39]. In addition to single administration studies, ketamine studies included in our study also included a considerable number of repeated infusion studies. Most of these studies had a treatment endpoint of about 13 days, which may be the main reason why the treatment results of 13 days were significantly better than those of 26 days. Second, it is possible that some patients relapsed after discontinuation of the drug. The study showed that repeated infusion may also prolong the time for depression relapse. The median time for relapse after the last ketamine infusion was about 18 days, and the level of suicidal ideation in patients was likely to change with the recurrence of depression.

There are also some limitations in our study. First of all, since there is no gold standard for the evaluation of suicidal ideation, most researchers adopt different methods for the diagnosis and evaluation of suicidal ideation, which also limits the integration of these research data and may also bring some bias to our research. Secondly, The sample size of the individual studies we included was low, which could also easily bias the results. As the study has been further processed by multi-arm research transformation and contains data of multiple time nodes, the sample size of some studies has been used several times, and the number of participants has been enlarged, which may have a certain impact on our study. However, we do not believe that this is a completely negative effect. The amplification of sample size may also make our network model more robust. In addition, besides the two-arm study, our NMA also includes three-arm and four-arm studies, which has a certain impact on the inspection and verification of the inconsistency of NMA and makes it very difficult to define the inconsistency. We did not do a good job of verifying that our NMA was inconsistent. The difficulty in defining inconsistency comes when we have 2- and 3-arm trial evidence, for example, AB, AC, BC, and ABC trials. Because inconsistency degrees of freedom (ICDF) corresponds to the number of independent loops, if a loop is formed from a multiarm trial alone, it is not counted as an independent loop and must therefore be discounted from the total ICDF. Thus, where there are mixtures of 2-arm and multiarm trials, our definition of inconsistency as arising in loops creates inherent technical difficulties that cannot, as far as is known, be avoided [40]. Observing through the funnel plot of the NMA, we consider its associated bias to be acceptable. We evaluated the randomized controlled trials included in (RoB 2). The results of our rigorous assessment of the risk of bias are shown in Supplementary Tables 1 and 2, and the overall bias of RCT studies is shown in Supplementary Fig. 7A, B. Two of the 10 studies on esketamine were from Janssen Pharmaceutical Companies, which may have created industry bias. All risk of bias was assessed by two study authors independently. If there were discrepancies between the two study authors, a third study author intervened to reassess the risk of bias. The opinions of all evaluated study authors were combined and the final decision was made by vote. We declare that none of the authors on our team have any conflicts of interest with ketamine products. For single-arm studies, we applied the MINORS. In this scale assessment, most of the information in the included articles was clearly reported and adequate. Little information reported but inadequate. Finally, we have to state that all the esketamine studies we included were from the same company, there was a lack of comparative studies from other institutions, and the number of studies was small. We remain suspicious of the results related to esketamine for the time being, which needs to be verified in more studies in the future.

The method of our NMA is innovative. It was an exploratory attempt. Although we cannot prove whether this method is reasonable enough at present, and many problems need to be further proved and explored in the future, it provides a new way of thinking for future NMA methods. Considering many aspects, we cannot completely rule out the existence of bias.

The purpose of our study was to explore the effect of ketamine on suicidal ideation in depressed patients. Our NMA provided a good explanation for the magnitude of reduction in suicidal ideation at different time points after ketamine use. This is of great reference value in how to use drugs more reasonably in clinical practice.

Ketamine’s advantage in rapidly alleviating suicidal ideation in patients with depression is consistent with previous studies [41, 42]. Our study provides strong research evidence to further support this research view. The rapid effect of ketamine in alleviating suicidal ideation may have important clinical implications, which can be the difference between life and death in suicidally depressed patients. The importance of long-term repeated ketamine administration is also illustrated in our study. This is of great significance for future drug development and clinical use.

### Supplementary information

PRISMA_2020_checklist

Supplementary Appendix

## References

[1] WHO. Preventing Suicide, A Global Imperative. Geneva, Switzerland: World Health Organization; 2014.

[2] Swannell SV, Martin GE, Page A, Hasking P, St John NJ. Prevalence of nonsuicidal self-injury in nonclinical samples: systematic review, meta-analysis and meta-regression. Suicide Life-threatening Behav. 2014;44:273–303. 10.1111/sltb.12070 24422986

[3] Hedegaard H, Curtin SC, Warner M. Increase in Suicide Mortality in the United States, 1999-2018. NCHS data brief. 2020:1-8. 32487287

[4] Ting SA, Sullivan AF, Boudreaux ED, Miller I, Camargo CA Jr. Trends in US emergency department visits for attempted suicide and self-inflicted injury, 1993-2008. Gen Hosp Psychiatry. 2012;34:557–65. 22554432 10.1016/j.genhosppsych.2012.03.020 PMC3428496

[5] Trivedi MH, Rush AJ, Wisniewski SR, Nierenberg AA, Warden D, Ritz L, et al. Evaluation of outcomes with citalopram for depression using measurement-based care in STARD: implications for clinical practice. Am J Psychiatry. 2006;163:28–40. 16390886 10.1176/appi.ajp.163.1.28

[6] Umetsu R, Abe J, Ueda N, Kato Y, Matsui T, Nakayama Y, et al. Association between selective serotonin reuptake inhibitor therapy and suicidality: analysis of U.S. Food and Drug Administration adverse event reporting system data. Biol Pharm Bull. 2015;38:1689–99. 26521821 10.1248/bpb.b15-00243

[7] McIntyre RS, Rodrigues NB, Lee Y, Lipsitz O, Subramaniapillai M, Gill H, et al. The effectiveness of repeated intravenous ketamine on depressive symptoms, suicidal ideation and functional disability in adults with major depressive disorder and bipolar disorder: results from the Canadian Rapid Treatment Center of Excellence. J Affect Disord. 2020;274:903–10. 32664031 10.1016/j.jad.2020.05.088

[8] Kheirabadi D, Kheirabadi GR, Mirlohi Z, Tarrahi MJ, Norbaksh A. Comparison of rapid antidepressant and antisuicidal effects of intramuscular ketamine, oral ketamine, and electroconvulsive therapy in patients with major depressive disorder: a pilot study. J Clin Psychopharmacol. 2020;40:588–93. 33060432 10.1097/JCP.0000000000001289

[9] Grunebaum MF, Galfalvy HC, Choo TH, Keilp JG, Moitra VK, Parris MS, et al. Ketamine for rapid reduction of suicidal thoughts in major depression: a midazolam-controlled randomized clinical trial. Am J Psychiatry. 2018;175:327–35. 29202655 10.1176/appi.ajp.2017.17060647 PMC5880701

[10] Chen Q, Dong J, Luo J, Ren L, Min S, Hao X, et al. Effects of low-dose ketamine on the antidepressant efficacy and suicidal ideations in patients undergoing electroconvulsive therapy. J ECT. 2020;36:25–30. 31913927 10.1097/YCT.0000000000000636

[11] Corwell BN, Motov SM, Davis NL, Kim HK. Novel uses of ketamine in the emergency department. Expert Opin Drug Saf. 2022;21:1009–25. 35822534 10.1080/14740338.2022.2100883

[12] Alnefeesi Y, Chen-Li D, Krane E, Jawad MY, Rodrigues NB, Ceban F, et al. Real-world effectiveness of ketamine in treatment-resistant depression: a systematic review & meta-analysis. J Psychiatr Res. 2022;151:693–709. 35688035 10.1016/j.jpsychires.2022.04.037

[13] Gardner DM, Murphy AL, O’Donnell H, Centorrino F, Baldessarini RJ. International consensus study of antipsychotic dosing. Am J Psychiatry. 2010;167:686–93. 20360319 10.1176/appi.ajp.2009.09060802

[14] Watt J, Del Giovane C. Network meta-analysis. Methods Mol Biol. 2022;2345:187–201. 34550592 10.1007/978-1-0716-1566-9_12

[15] Chaimani A, Higgins JP, Mavridis D, Spyridonos P, Salanti G. Graphical tools for network meta-analysis in STATA. PLoS ONE. 2013;8:e76654. 24098547 10.1371/journal.pone.0076654 PMC3789683

[16] Sterne JAC, Savović J, Page MJ, Elbers RG, Blencowe NS, Boutron I, et al. RoB 2: a revised tool for assessing risk of bias in randomised trials. BMJ (Clin Res ed). 2019;366:l4898. 10.1136/bmj.l4898 31462531

[17] Slim K, Nini E, Forestier D, Kwiatkowski F, Panis Y, Chipponi J. Methodological index for non-randomized studies (minors): development and validation of a new instrument. ANZ J Surg. 2003;73:712–6. 12956787 10.1046/j.1445-2197.2003.02748.x

[18] Mbuagbaw L, Rochwerg B, Jaeschke R, Heels-Andsell D, Alhazzani W, Thabane L, et al. Approaches to interpreting and choosing the best treatments in network meta-analyses. Syst Rev. 2017;6:79. 28403893 10.1186/s13643-017-0473-z PMC5389085

[19] Braun C, Bschor T, Franklin J, Baethge C. Suicides and suicide attempts during long-term treatment with antidepressants: a meta-analysis of 29 placebo-controlled studies including 6,934 patients with major depressive disorder. Psychother Psychosom. 2016;85:171–9. 27043848 10.1159/000442293

[20] Guzzetta F, Tondo L, Centorrino F, Baldessarini RJ. Lithium treatment reduces suicide risk in recurrent major depressive disorder. J Clin Psychiatry. 2007;68:380. 17388706 10.4088/JCP.v68n0304

[21] Turner BJ, Austin SB, Chapman AL. Treating Nonsuicidal Self-Injury: A Systematic Review of Psychological and Pharmacological Interventions. Los Angeles, CA: SAGE Publications; 2014. 576-85. 10.1177/070674371405901103 PMC4244876 25565473

[22] Lengvenyte A, Olié E, Courtet P. Suicide has many faces, so does ketamine: a narrative review on ketamine’s antisuicidal actions. Curr Psychiatry Rep. 2019;21:132–10. 31797066 10.1007/s11920-019-1108-y

[23] Huang X, Harris LM, Funsch KM, Fox KR, Ribeiro JD. Efficacy of psychotropic medications on suicide and self-injury: a meta-analysis of randomized controlled trials. Transl Psychiatry. 2022;12:400. 36130938 10.1038/s41398-022-02173-9 PMC9492722

[24] Gilbert JR, Gerner JL, Burton CR, Nugent AC, Zarate CA Jr., Ballard ED. Magnetoencephalography biomarkers of suicide attempt history and antidepressant response to ketamine in treatment-resistant major depression. J Affect Disord. 2022;312:188–97. 35728680 10.1016/j.jad.2022.06.025 PMC9262873

[25] Keilp JG, Madden SP, Marver JE, Frawley A, Burke AK, Herzallah MM, et al. Effects of ketamine versus midazolam on neurocognition at 24 h in depressed patients with suicidal ideation. J Clin Psychiatry. 2021;82:21m13921. 34727422 10.4088/JCP.21m13921

[26] Fava M, Freeman MP, Flynn M, Judge H, Hoeppner BB, Cusin C, et al. Double-blind, placebo-controlled, dose-ranging trial of intravenous ketamine as adjunctive therapy in treatment-resistant depression (TRD). Mol Psychiatry. 2020;25:1592–603. 30283029 10.1038/s41380-018-0256-5 PMC6447473

[27] Geisslinger G, Hering W, Thomann P, Knoll R, Kamp HD, Brune K. Pharmacokinetics and pharmacodynamics of ketamine enantiomers in surgical patients using a stereoselective analytical method. Br J Anaesth. 1993;70:666–71. 8329260 10.1093/bja/70.6.666

[28] Zarate CA Jr., Brutsche N, Laje G, Luckenbaugh DA, Venkata SL, Ramamoorthy A, et al. Relationship of ketamine’s plasma metabolites with response, diagnosis, and side effects in major depression. Biol Psychiatry. 2012;72:331–8. 22516044 10.1016/j.biopsych.2012.03.004 PMC3442255

[29] Clements JA, Nimmo WS, Grant IS. Bioavailability, pharmacokinetics, and analgesic activity of ketamine in humans. J Pharm Sci. 1982;71:539–42. 7097501 10.1002/jps.2600710516

[30] Malinovsky JM, Servin F, Cozian A, Lepage JY, Pinaud M. Ketamine and norketamine plasma concentrations after i.v., nasal and rectal administration in children. Br J Anaesth. 1996;77:203–7. 8881626 10.1093/bja/77.2.203

[31] Rolan P, Lim S, Sunderland V, Liu Y, Molnar V. The absolute bioavailability of racemic ketamine from a novel sublingual formulation. Br J Clin Pharmacol. 2014;77:1011–6. 24977293 10.1111/bcp.12264 PMC4093926

[32] Lee EE, Della Selva MP, Liu A, Himelhoch S. Ketamine as a novel treatment for major depressive disorder and bipolar depression: a systematic review and quantitative meta-analysis. Gen Hosp Psychiatry. 2015;37:178–84. 25698228 10.1016/j.genhosppsych.2015.01.003

[33] Kishimoto T, Chawla JM, Hagi K, Zarate CA, Kane JM, Bauer M, et al. Single-dose infusion ketamine and non-ketamine N-methyl-d-aspartate receptor antagonists for unipolar and bipolar depression: a meta-analysis of efficacy, safety and time trajectories. Psychol Med. 2016;46:1459–72. 26867988 10.1017/S0033291716000064 PMC5116384

[34] Romeo B, Choucha W, Fossati P, Rotge JY. Meta-analysis of short- and mid-term efficacy of ketamine in unipolar and bipolar depression. Psychiatry Res. 2015;230:682–8. 26548981 10.1016/j.psychres.2015.10.032

[35] Zheng W, Zhou YL, Liu WJ, Wang CY, Zhan YN, Li HQ, et al. Rapid and longer-term antidepressant effects of repeated-dose intravenous ketamine for patients with unipolar and bipolar depression. J Psychiatr Res. 2018;106:61–8. 30278319 10.1016/j.jpsychires.2018.09.013

[36] Murrough JW, Perez AM, Pillemer S, Stern J, Parides MK, aan het Rot M, et al. Rapid and longer-term antidepressant effects of repeated ketamine infusions in treatment-resistant major depression. Biol Psychiatry. 2013;74:250–6. 22840761 10.1016/j.biopsych.2012.06.022 PMC3725185

[37] aan het Rot M, Collins KA, Murrough JW, Perez AM, Reich DL, Charney DS, et al. Safety and efficacy of repeated-dose intravenous ketamine for treatment-resistant depression. Biol Psychiatry. 2010;67:139–45. 19897179 10.1016/j.biopsych.2009.08.038

[38] Zhao X, Venkata SL, Moaddel R, Luckenbaugh DA, Brutsche NE, Ibrahim L, et al. Simultaneous population pharmacokinetic modelling of ketamine and three major metabolites in patients with treatment-resistant bipolar depression. Br J Clin Pharmacol. 2012;74:304–14. 22295895 10.1111/j.1365-2125.2012.04198.x PMC3630750

[39] Adamowicz P, Kala M. Urinary excretion rates of ketamine and norketamine following therapeutic ketamine administration: method and detection window considerations. J Anal Toxicol. 2005;29:376–82. 16105264 10.1093/jat/29.5.376

[40] Dias S, Welton NJ, Sutton AJ, Caldwell DM, Lu G, Ades AE. Evidence synthesis for decision making 4: inconsistency in networks of evidence based on randomized controlled trials. Med Decis Mak. 2013;33:641–56. 10.1177/0272989X12455847 PMC3704208 23804508

[41] Jawad MY, Di Vincenzo JD, Badulescu S, Teopiz KM, Tabassum A, Ceban F, et al. The therapeutic role of ketamine and esketamine in treating psychopathological domains of depression. Neuropharmacology. 2022;223:109299. 36336068 10.1016/j.neuropharm.2022.109299

[42] Alario AA, Niciu MJ. (Es)Ketamine for Suicidal Ideation and Behavior: Clinical Efficacy. Los Angeles, CA: SAGE Publications; 2022. 10.1177/24705470221128017 PMC9585565 36276228

[43] Zheng W, Gu LM, Zhou YL, Wang CY, Lan XF, Zhang B, et al. Plasma VEGF concentrations and ketamine’s effects on suicidal ideation in depression with suicidal ideation. Front Psychiatry. 2022;13:855995. 35546941 10.3389/fpsyt.2022.855995 PMC9084596

[44] Abbar M, Demattei C, El-Hage W, Llorca PM, Samalin L, Demaricourt P, et al. Ketamine for the acute treatment of severe suicidal ideation: double blind, randomised placebo controlled trial. BMJ (Clin Res ed). 2022;376:e067194. 10.1136/bmj-2021-067194 PMC8808464 35110300

[45] Feeney A, Hock RS, Freeman MP, Flynn M, Hoeppner B, Iosifescu DV, et al. The effect of single administration of intravenous ketamine augmentation on suicidal ideation in treatment-resistant unipolar depression: Results from a randomized double-blind study. Eur Neuropsychopharmacol. 2021;49:122–32. 34090255 10.1016/j.euroneuro.2021.04.024 PMC8338746

[46] Domany Y, McCullumsmith CB. Single, fixed-dose intranasal ketamine for alleviation of acute suicidal ideation. an emergency department, trans-diagnostic approach: a randomized, double-blind, placebo-controlled, proof-of-concept trial. Arch Suicide Res. 2022;26:1250–65. 33583341 10.1080/13811118.2021.1878078

[47] Canuso CM, Ionescu DF, Li X, Qiu X, Lane R, Turkoz I, et al. Esketamine nasal spray for the rapid reduction of depressive symptoms in major depressive disorder with acute suicidal ideation or behavior. J Clin Psychopharmacol. 2021;41:516–24. 34412104 10.1097/JCP.0000000000001465 PMC8407443

[48] Zhou Y, Liu W, Zheng W, Wang C, Zhan Y, Lan X, et al. Predictors of response to repeated ketamine infusions in depression with suicidal ideation: an ROC curve analysis. J Affect Disord. 2020;264:263–71. 32056760 10.1016/j.jad.2020.01.001

[49] Canuso CM, Singh JB, Fedgchin M, Alphs L, Lane R, Lim P, et al. Efficacy and safety of intranasal esketamine for the rapid reduction of symptoms of depression and suicidality in patients at imminent risk for suicide: results of a double-blind, randomized, placebo-controlled study. Am J Psychiatry. 2018;175:620–30. 29656663 10.1176/appi.ajp.2018.17060720

[50] Price RB, Iosifescu DV, Murrough JW, Chang LC, Al Jurdi RK, Iqbal SZ, et al. Effects of ketamine on explicit and implicit suicidal cognition: a randomized controlled trial in treatment-resistant depression. Depress Anxiety. 2014;31:335–43. 24668760 10.1002/da.22253 PMC4112410

[51] Ionescu DF, Bentley KH, Eikermann M, Taylor N, Akeju O, Swee MB, et al. Repeat-dose ketamine augmentation for treatment-resistant depression with chronic suicidal ideation: a randomized, double blind, placebo controlled trial. J Affect Disord. 2019;243:516–24. 30286416 10.1016/j.jad.2018.09.037

[52] Domany Y, Shelton RC, McCullumsmith CB. Ketamine for acute suicidal ideation. An emergency department intervention: a randomized, double-blind, placebo-controlled, proof-of-concept trial. Depress Anxiety. 2020;37:224–33. 31733088 10.1002/da.22975

[53] Burger J, Capobianco M, Lovern R, Boche B, Ross E, Darracq MA, et al. A double-blinded, randomized, placebo-controlled sub-dissociative dose ketamine pilot study in the treatment of acute depression and suicidality in a military emergency department setting. Mil Med. 2016;181:1195–9. 27753551 10.7205/MILMED-D-15-00431

[54] Zhan Y, Zhang B, Zhou Y, Zheng W, Liu W, Wang C, et al. A preliminary study of anti-suicidal efficacy of repeated ketamine infusions in depression with suicidal ideation. J Affect Disord. 2019;251:205–12. 30927581 10.1016/j.jad.2019.03.071

