# Correction: A meta-analysis of the effects of ketamine on suicidal ideation in depression patients

**DOI:** 10.1038/s41398-024-02979-9

**Published:** 2024-07-23

**Authors:** ZuoYao Shen, DaiQuan Gao, Xue Lv, HongXing Wang, WeiHua Yue

**Affiliations:** 1 The First Affiliated Hospital of Xinxiang Medical College Henan, Henan, China; 2 Peking University Sixth Hospital, Peking University Institute of Mental Health, Beijing, China; 3 NHC Key Laboratory of Mental Health (Peking University), National Clinical Research Center for Mental Disorders (Peking University Sixth Hospital), Beijing, China; 4 Research Unit of Diagnosis and Treatment of Mood Cognitive Disorder, Chinese Academy of Medical Sciences (No.2018RU006), Beijing, China; 5 Department of Neurology, Xuanwu Hospital, Capital Medical University, Beijing, China

**Keywords:** Clinical pharmacology, Depression

Correction to: *Translational Psychiatry* 10.1038/s41398-024-02973-1, published online 10 June 2024

In Table 1 of this article, the column headed year was not wide enough to show the exact year.

Table 1 should appear like that:

**Table:** 

Study	Year	Race	Method	Frequency	Primary intervention	Blind assessment	Type of patients	Type of treatment setting	Type of effect	Sample size	Age (years) Mean/median (range)	Male (%)	Trial duration	Diagnostic tools	Suicidal ideation remission definition	Ketamine dosage
Wei Zheng [43]	2022	Asian	Intravenous	Repeatedly	Ketamine	Single arm	MDD or BD	Inpatient	Long term duration	60	35.3 (12.4)	27 (45%)	12 days	DSM-V	SSI part I <2	0.5 mg/kg
Mocrane Abbar [44]	2021	European	Intravenous	Repeatedly	Ketamine/Placebo	Double-Blinded	Bipolar, Depressive	Inpatient	Acute	73/83	38 (18-75)/41 (18-76)	19 (26.0)/31 (37.3)	6 weeks	DSM-IV	SSI score of 0 to 3	0.5 mg/kg
Anna Feeney [45]	2021	American	Intravenous	Single	Ketamine/Midazolam	Double-Blinded	MDD	Outpatients	Long term duration	40/16	45.75±12.32	28 (50%)	3 days	DSMIV-TR TM	MADRS suicide item < 2	0.1 mg/kg, 0.5 mg/kg, 1.0 mg/kg
Yoav Domany [46]	2021	American	Intranasal	Ringle	Ketamine/Placebo	Double-Blinded	Mental disorders	Inpatient	Acute	15/15	35.11 (8.67)/35.78 (9.86)	4 (44.4%) /4 (44.4%)	4 hours	NR	MADRS suicide item = 0	40 mg
Carla M. Canuso [47]	2021	American	Intranasal	Repeatedly	Esketamine/Placebo	Double-Blinded	MDD	Inpatient	Acute	222/224	40.5 (12.92) /39.6 (13.08)	92 (40.7) /85 (37.8)	4 weeks	DSM-V	CGI-SS-r score of 0 to 1	84 mg
Yanling Zhou [48]	2020	Asian	Intravenous	Repeatedly	Ketamine	Single arm	Depression	Inpatient	Long term duration	73	35.2(11.8)	35(47.9)	12 days	DSM-V	SSI part I <2	0.5 mg/kg
Carla M. Canuso [49]	2018	American	Intranasal	Repeatedly	Esketamine/Placebo	Double-Blinded	Major depression	Inpatient	Acute	35/31	35.7 (13.40) /36.0 (12.82)	13 (37.1) /10 (32.3)	4 weeks	DSM-IV-TR	CGI-SS-r score of 0 to 1	84 mg
Roger S McIntyre [7)	2020	American	Intravenous	Repeatedly	Ketamine	Single arm	MDD or BD	Outpatients	Long term duration	213	45 (15)	95 (44.6)	2 weeks	DSM-V	QIDS-SR16 = 0	0.5 ~ 0.75 mg/kg
Rebecca B. Price [50]	2014	American	Intravenous	Single	Ketamine/Midazolam	Double-Blinded	TRD	Outpatients	Acute	36/21	48.6 (11.4)/43.8 (10.9)	16 (44%)/11 (52%)	24 hours	DSM-IV-TR	QIDS-SR16 = 0	0.5 mg/kg
Dawn F. Ionescu [51)	2019	American	Intravenous	Repeatedly	Ketamine/Placebo	Double-Blinded	MDD	Outpatients	Long term duration	13/13	45.5 (13.6) /45.3 (11.7)	6 (46%) /10 (77%)	3 weeks	DSM-IV	C-SSRS SI score = 0	0.5 mg/kg
Yoav Domany [52)	2019	American	Intravenous	Single	Ketamine/Placebo	Double-Blinded	MDD, BD, Depression, Dysthymia	Inpatient	Acute	9/9	35.11 (8.67)/35.78 (9.86)	4 (44.4%)/4 (44.4%)	24 hours	MINI	MADRS suicide item score of 0 to 2	0.2 mg/kg
Michael F. Grunebaum [9]	2018	American	Intravenous	Single	Ketamine/Midazolam	Double-Blinded	MDD	Inpatient	Acute	40/40	38.4 (13.2)/40.7 (13.1)	18(45%)/14 (35%)	24 hours	DSM-IV	SSI <3	0.5 mg/kg
LT John Burger [53]	2016	American	Intravenous	Single	Ketamine/Placebo	Double-Blinded	Depression	Inpatient	Acute	3/7	28/27	2 (67%)/5 (71%)	4 hours	NR	SSI score of 0 to 3	0.2 mg/kg
Zhan Yanni [54]	2019	Asian	Intravenous	Repeatedly	Ketamine	Single arm	Depression	Inpatient	Long term duration	86	33.99 (11.65)	43 (50.0%)	12 days	DSM-V (SCID-5)	SSI part I <2	0.5 mg/kg

The original article has been corrected.

