# Supplementary material for: A meta-analysis of the effects of ketamine on suicidal ideation in depression patients
Source: Transl Psychiatry. 2024 Jun 10;14:248. doi: 10.1038/s41398-024-02973-1 (PMC11164699; doi:10.1038/s41398-024-02973-1)
Supplement: Supplementary file 1 — PRISMA_2020_checklist [file 41398_2024_2973_MOESM1_ESM.pdf]

PRISMA 2020 Checklist

Section and Topic	Item #	Checklist item	Location where item is reported
**TITLE**			
Title	1	Identify the report as a systematic review.	The title is stated as a Meta-analysis.
**ABSTRACT**			
Abstract	2	See the PRISMA 2020 for Abstracts checklist.	Abstracts have been described as requested.
**INTRODUCTION**			
Rationale	3	Describe the rationale for the review in the context of existing knowledge.	Rationale is described in lines 47 to 64.
Objectives	4	Provide an explicit statement of the objective(s) or question(s) the review addresses.	Objectives is described in lines 65 to 71.
**METHODS**			
Eligibility criteria	5	Specify the inclusion and exclusion criteria for the review and how studies were grouped for the syntheses.	Eligibility criteria is described in lines 89 to 105.
Information sources	6	Specify all databases, registers, websites, organisations, reference lists and other sources searched or consulted to identify studies. Specify the date when each source was last searched or consulted.	Information sources is described in lines 83 to 87.
Search strategy	7	Present the full search strategies for all databases, registers and websites, including any filters and limits used.	The search strategy is described at the end of the Supplementary Appendix.
Selection process	8	Specify the methods used to decide whether a study met the inclusion criteria of the review, including how many reviewers screened each record and each report retrieved, whether they worked independently, and if applicable, details of automation tools used in the process.	The Selection process section is described in lines 89 to 105.
Data collection process	9	Specify the methods used to collect data from reports, including how many reviewers collected data from each report, whether they worked independently, any processes for obtaining or confirming data from study investigators, and if applicable, details of automation tools used in the process.	We describe the Data collection process in lines 120-134.
Data items	10a	List and define all outcomes for which data were sought. Specify whether all results that were compatible with each outcome domain in each study were sought (e.g. for all measures, time points, analyses), and if not, the methods used to decide which results to collect.	This section is answered in lines 120 to 125.
	10b	List and define all other variables for which data were sought (e.g. participant and intervention characteristics, funding sources). Describe any assumptions made about any missing or unclear information.	Lines 336 to 347 describe the content where this problem exists.
Study risk of bias assessment	11	Specify the methods used to assess risk of bias in the included studies, including details of the tool(s) used, how many reviewers assessed each study and whether they worked independently, and if applicable, details of automation tools used in the process.	Study risk of bias assessment is in lines 112-118.
Effect measures	12	Specify for each outcome the effect measure(s) (e.g. risk ratio, mean difference) used in the synthesis or presentation of results.	In lines 146 to 148 we describe the Effect measures.
Synthesis	13a	Describe the processes used to decide which studies were eligible for each synthesis (e.g. tabulating the study intervention characteristics and comparing against the planned groups for each synthesis (item #5)).	This section of information is described in Table 1.

methods	13b	Describe any methods required to prepare the data for presentation or synthesis, such as handling of missing summary statistics, or data conversions.	The process of transforming the data is described in lines 125 to 131.
	13c	Describe any methods used to tabulate or visually display results of individual studies and syntheses.	This information is described in lines 150-160.
	13d	Describe any methods used to synthesize results and provide a rationale for the choice(s). If meta-analysis was performed, describe the model(s), method(s) to identify the presence and extent of statistical heterogeneity, and software package(s) used.	This information is described in lines 142 to 160.
	13e	Describe any methods used to explore possible causes of heterogeneity among study results (e.g. subgroup analysis, meta-regression).	In lines 160, subgroup analyses were used.
	13f	Describe any sensitivity analyses conducted to assess robustness of the synthesized results.	In lines 157 to 160, we describe the sensitivity analysis methods.
Reporting bias assessment	14	Describe any methods used to assess risk of bias due to missing results in a synthesis (arising from reporting biases).	The bias assessment is described in lines 112 to 118.
Certainty assessment	15	Describe any methods used to assess certainty (or confidence) in the body of evidence for an outcome.	The bias assessment is described in lines 112 to 118.

PRISMA 2020 Checklist

Section and Topic	Item #	Checklist item	Location where item is reported
**RESULTS**			
Study selection	16a	Describe the results of the search and selection process, from the number of records identified in the search to the number of studies included in the review, ideally using a flow diagram.	The flow diagram is shown in figure1
	16b	Cite studies that might appear to meet the inclusion criteria, but which were excluded, and explain why they were excluded.	The reasons for exclusion are expressed in lines 165 to 168
Study characteristics	17	Cite each included study and present its characteristics.	The information for each study is described in Table 1
Risk of bias in studies	18	Present assessments of risk of bias for each included study.	The results of Risk of bias are presented in Supplementary Table 1,2 in the Supplementary Appendix
Results of individual studies	19	For all outcomes, present, for each study: (a) summary statistics for each group (where appropriate) and (b) an effect estimate and its precision (e.g. confidence/credible interval), ideally using structured tables or plots.	The Results of individual studies are shown in Figure 3,5
Results of syntheses	20a	For each synthesis, briefly summarise the characteristics and risk of bias among contributing studies.	The Results of individual studies are shown in Figure 2.
	20b	Present results of all statistical syntheses conducted. If meta-analysis was done, present for each the summary estimate and its precision (e.g. confidence/credible interval) and measures of statistical heterogeneity. If comparing groups, describe the direction of the effect.	The Results of individual studies are shown in Figure 3,5
	20c	Present results of all investigations of possible causes of heterogeneity among study results.	Lines 211-213 describe race as a possible cause of heterogeneity
	20d	Present results of all sensitivity analyses conducted to assess the robustness of the synthesized results.	This result is shown in Supplementary Figure 4,5,6 in the Supplementary Appendix
Reporting biases	21	Present assessments of risk of bias due to missing results (arising from reporting biases) for each synthesis assessed.	Lines 173-174 describe the risk of bias.
Certainty of evidence	22	Present assessments of certainty (or confidence) in the body of evidence for each outcome assessed.	Lines 183-191 describe the Certainty of evidence
**DISCUSSION**			
Discussion	23a	Provide a general interpretation of the results in the context of other evidence.	Lines 217 to 222 describe support from other evidence
	23b	Discuss any limitations of the evidence included in the review.	Lines 222 to 225describe the limitations of the review
	23c	Discuss any limitations of the review processes used.	Lines 310 to 347 describe the limitations of the study
	23d	Discuss implications of the results for practice, policy, and future research.	Lines 235 to 240 describe the impact of the study
**OTHER INFORMATION**			
Registration and protocol	24a	Provide registration information for the review, including register name and registration number, or state that the review was not registered.	The study was not registered
	24b	Indicate where the review protocol can be accessed, or state that a protocol was not prepared.	The study was not registered

	24c	Describe and explain any amendments to information provided at registration or in the protocol.	The study was not registered
Support	25	Describe sources of financial or non-financial support for the review, and the role of the funders or sponsors in the review.	We describe support in the ACKNOWLEDGEMENTS section
Competing interests	26	Declare any competing interests of review authors.	We made a statement in the COMPETING INTERESTS section
Availability of data, code and other materials	27	Report which of the following are publicly available and where they can be found: template data collection forms; data extracted from included studies; data used for all analyses; analytic code; any other materials used in the review.	All our information is public, and the data can be obtained in the relevant study, the relevant code can be found on the stata official website, and the tables are in the format required for software analysis

From: Page MJ, McKenzie JE, Bossuyt PM, Boutron I, Hoffmann TC, Mulrow CD, et al. The PRISMA 2020 statement: an updated guideline for reporting systematic reviews. BMJ 2021;372:n71. doi: 10.1136/bmj.n71
