# Supplementary material for: A meta-analysis of the effects of ketamine on suicidal ideation in depression patients
Source: Transl Psychiatry. 2024 Jun 10;14:248. doi: 10.1038/s41398-024-02973-1 (PMC11164699; doi:10.1038/s41398-024-02973-1)
Supplement: Supplementary file 2 — Supplementary Appendix [file 41398_2024_2973_MOESM2_ESM.docx]

**Supplementary Appendix**

**Supplementary Figures**

**Supplementary Figure 1**

**Example of data statistics process**


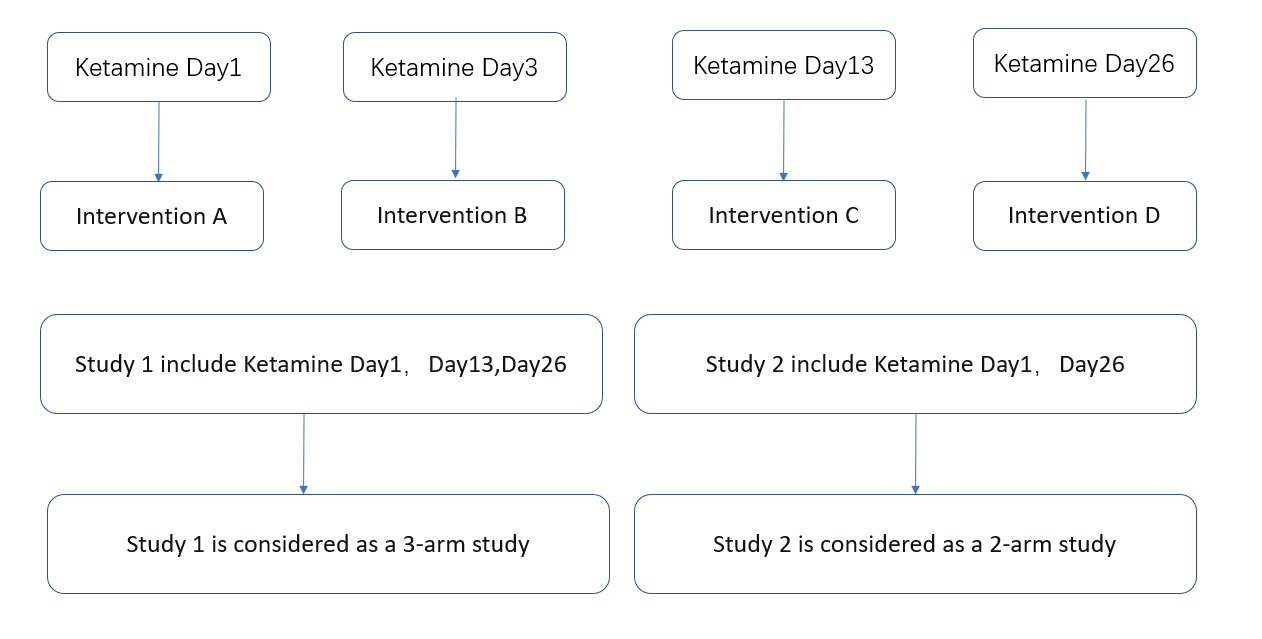


**Supplementary Figure 2**

**Cumulative probability of different treatment measures affecting the remission level of suicidal ideation under multiple time nodes**


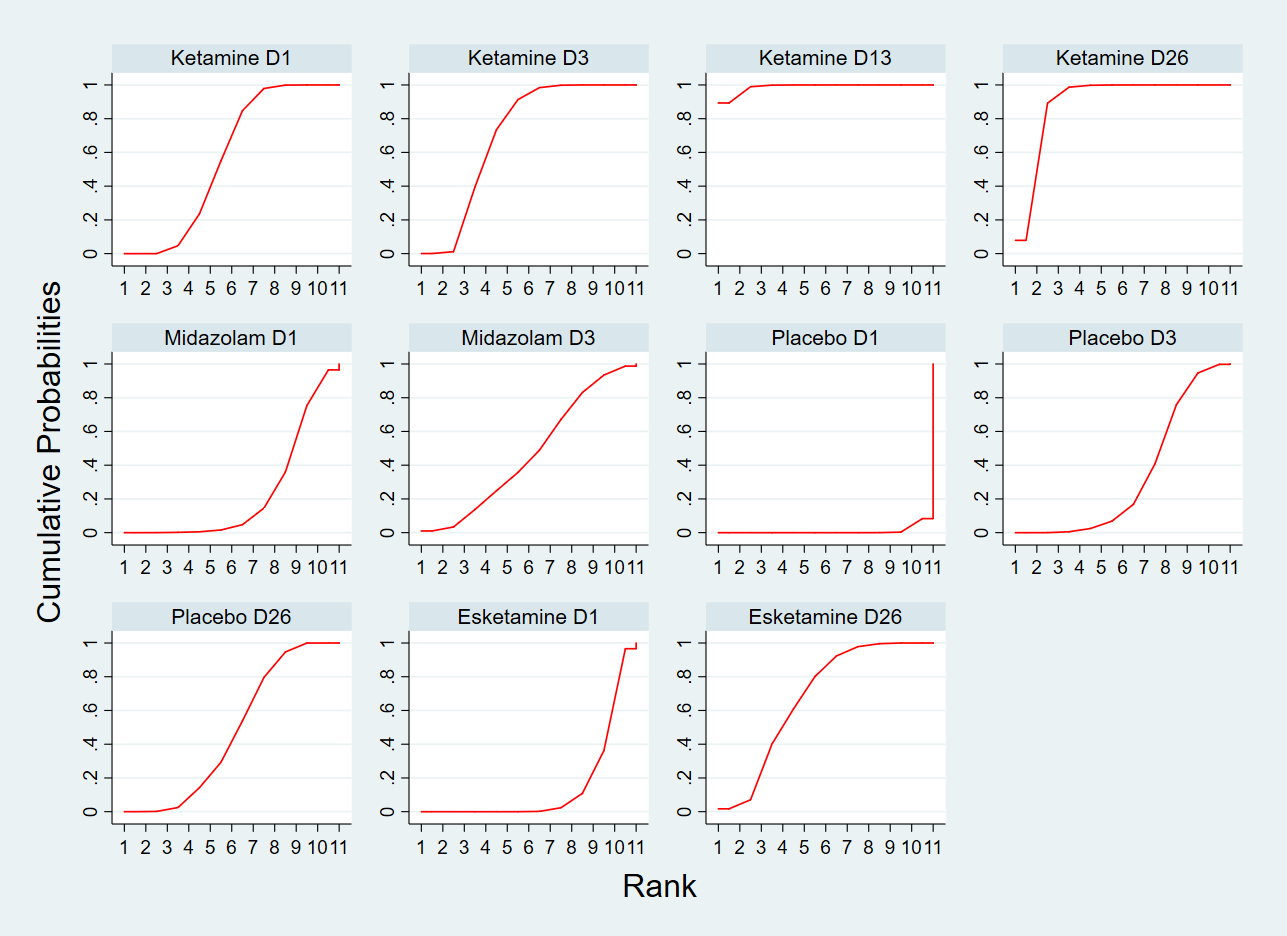


**Supplementary Figure 3**

**Contribution plot of network meta-analysis**


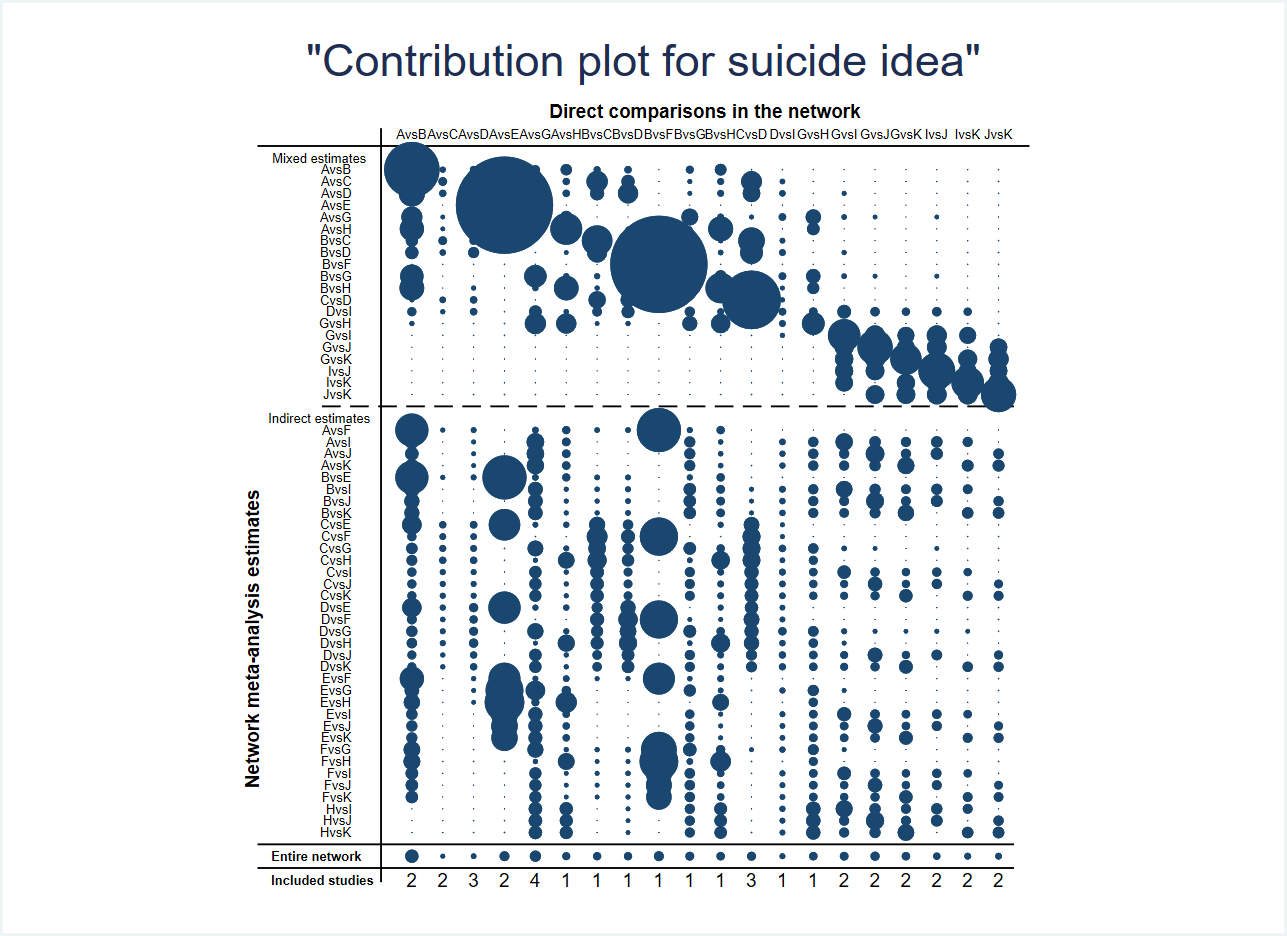


**Supplementary Figure 4**

**Funnel plot of network meta-analysis**

**
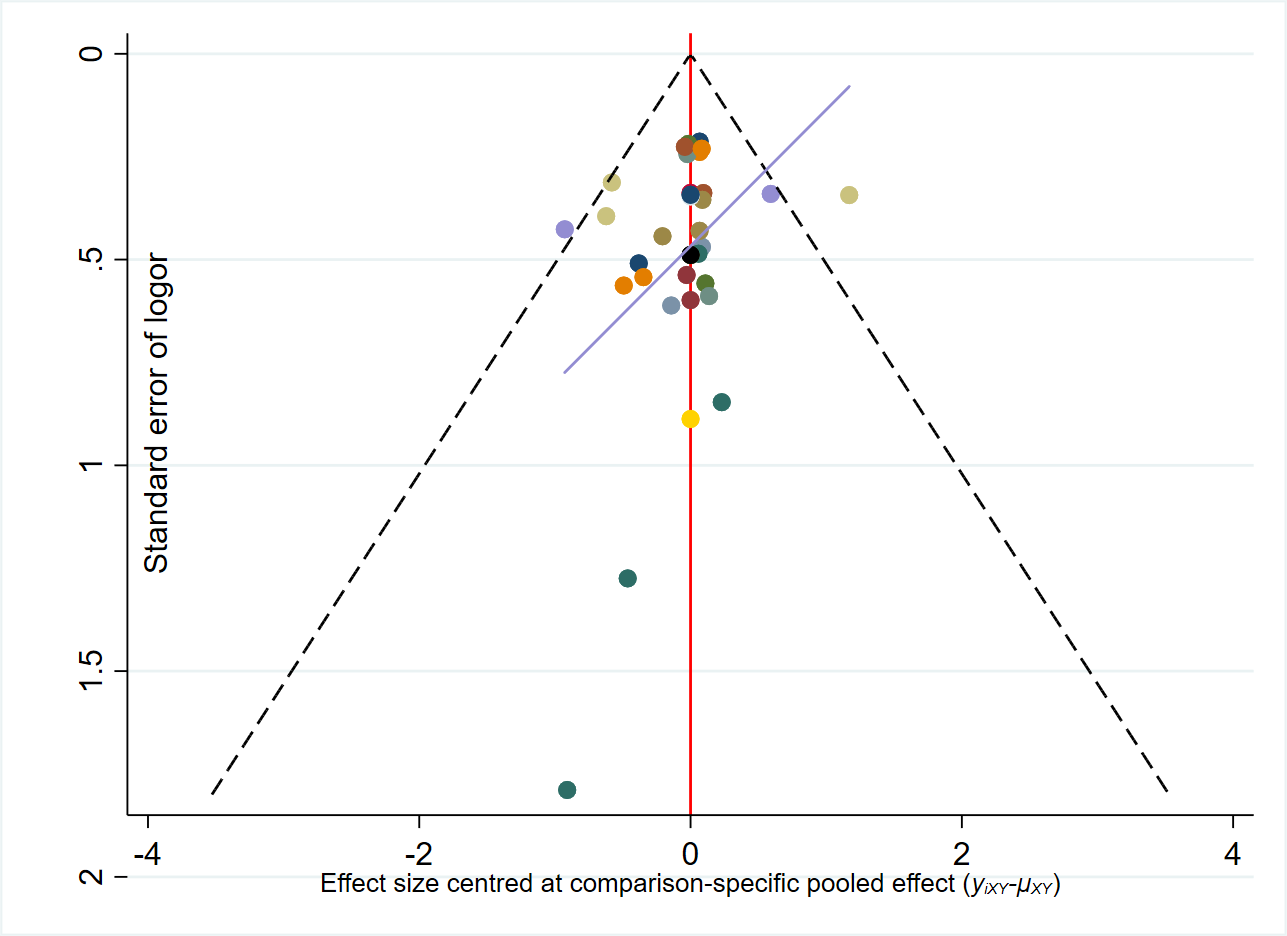
**

**Supplementary Figure 5**

**Egger’s publication bias plot of the remission effect of suicidal ideation between the first and the last treatment in the repeated administration group**


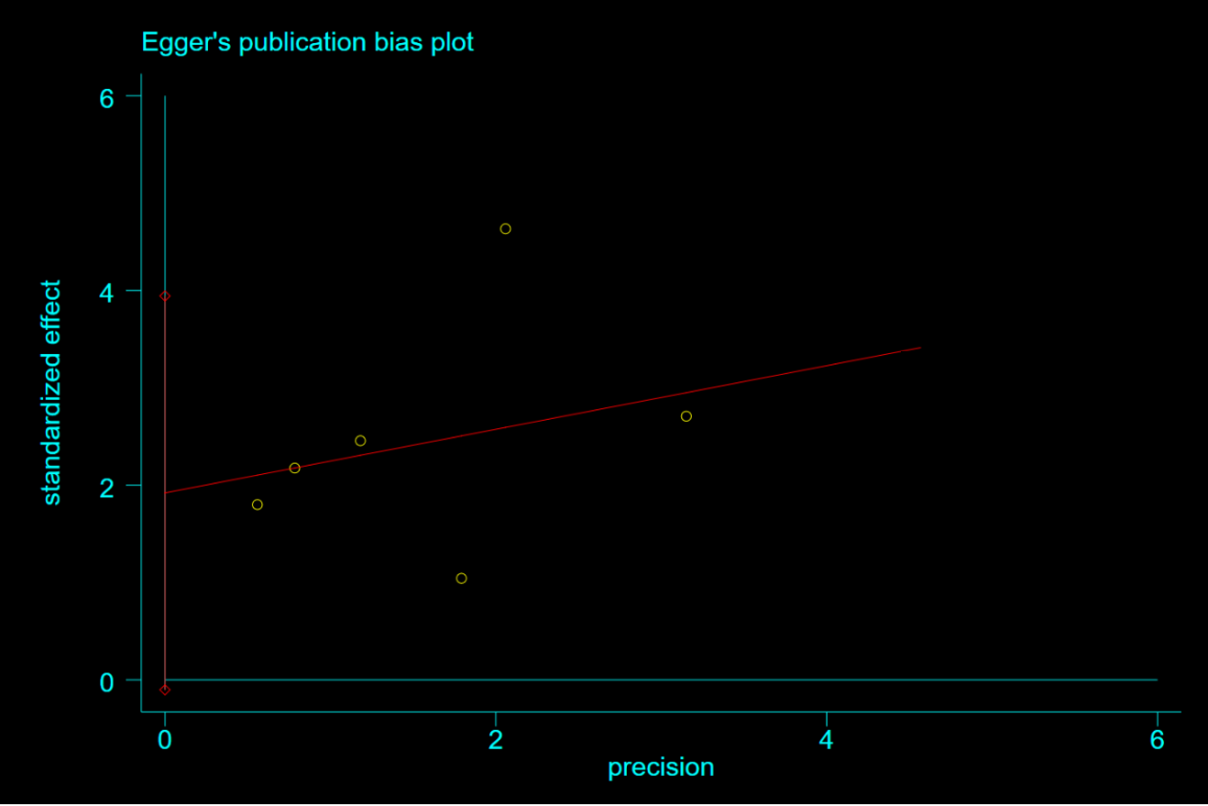


**Supplementary Figure 6**

**Funnel plot of the remission effect of suicidal ideation between the first and the last treatment in the repeated administration group**


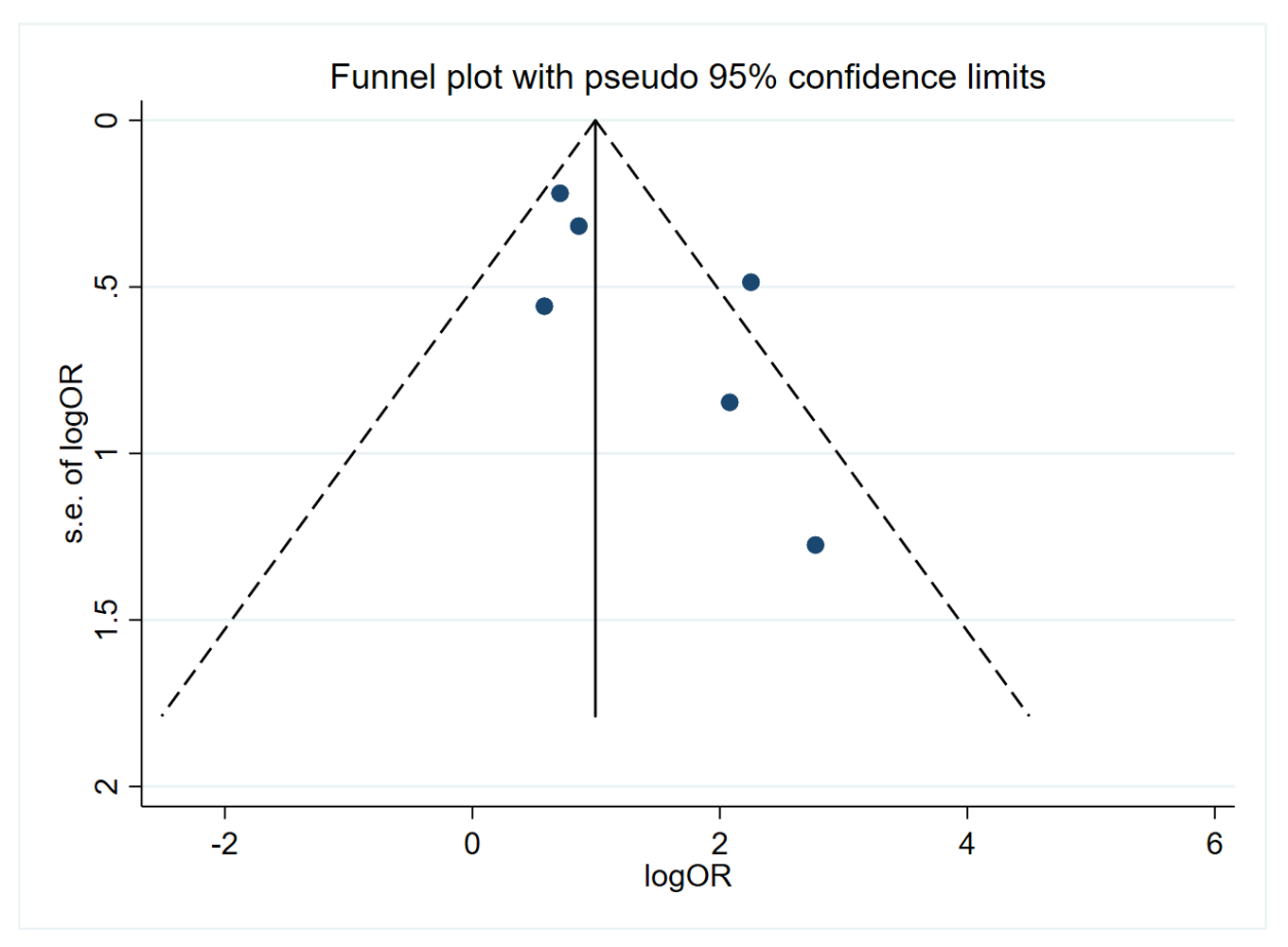


**Supplementary Figure 7**

**Supplementary Figure 7(A)**

**Risk of bias of included studies in meta-analysis of ketamine studies**


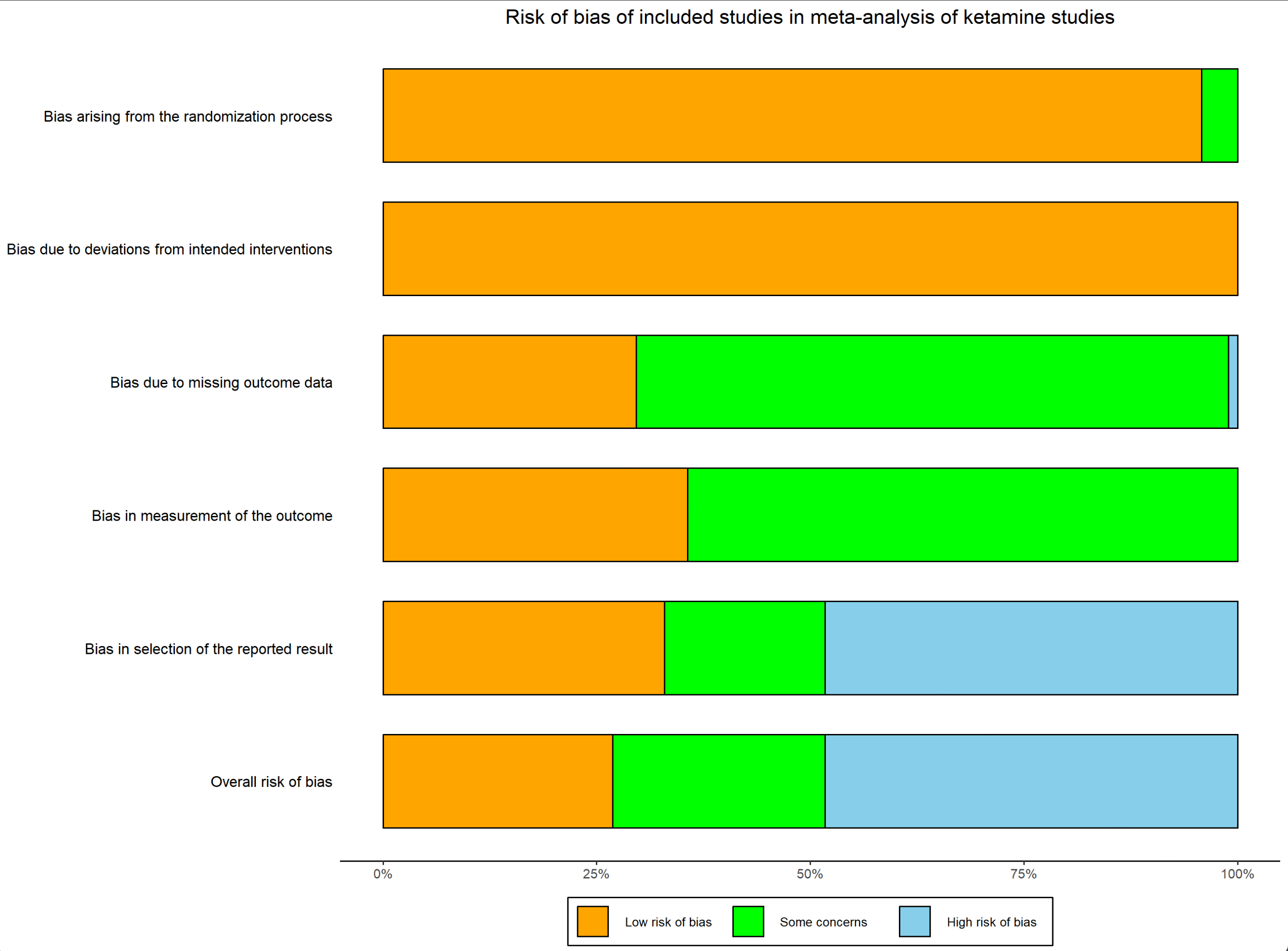


**Supplementary Figure 7(B)**

**Risk of bias of included studies in meta-analysis of ketamine studies**


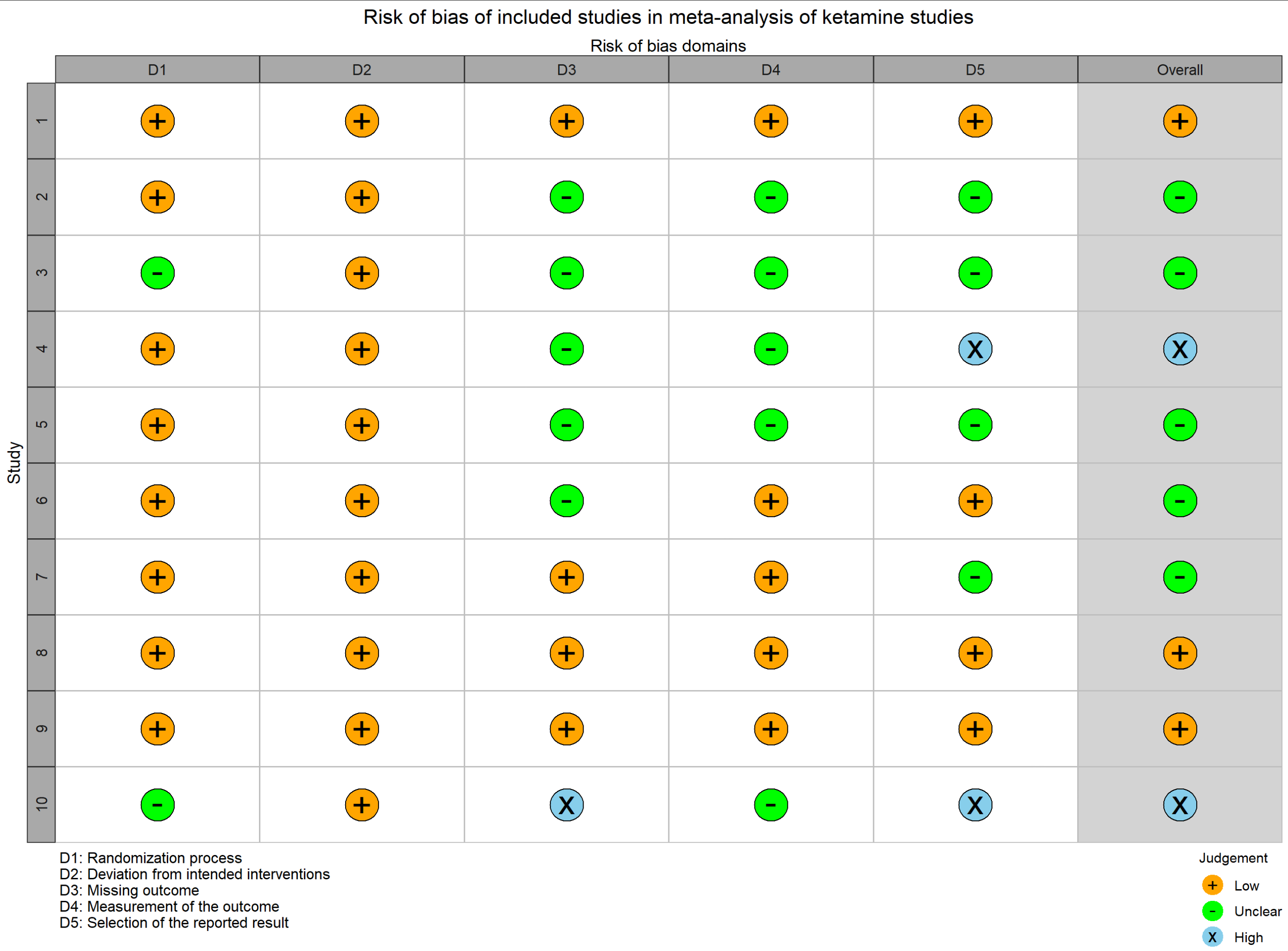


**Supplementary Tables**

**Supplementary Table 1. Risk of bias: RCT (ROB 2).**


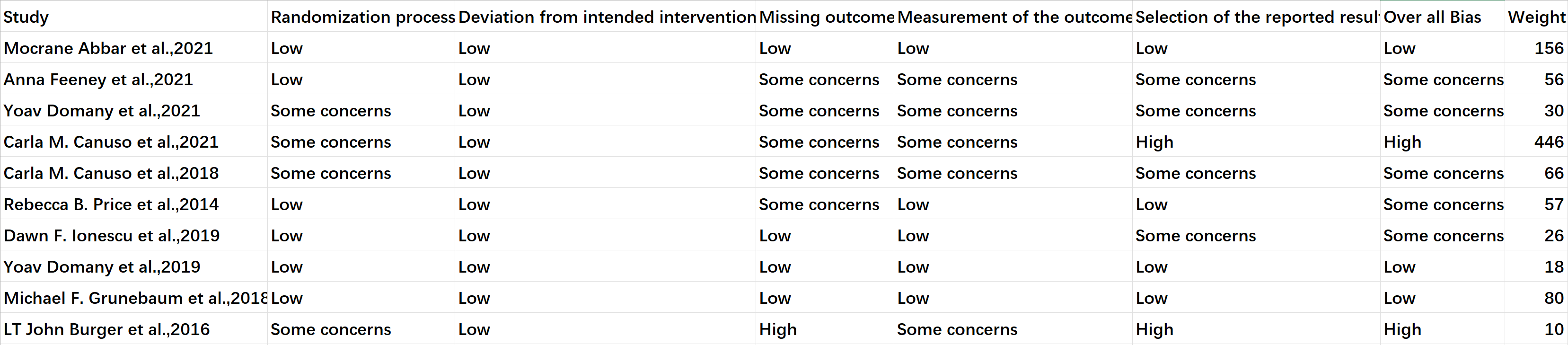


RoB 2, Cochrane Risk of Bias tool 2.

**Supplementary Table 2. Risk of bias: non-RCT (MINORS).**

MINORS, Methodological Index for Non-Randomized Studies.

0, not report; 1, report but inadequate; 2, Report and adequate.

**Supplementary Table 3. The probability ranking SUCRA of the effect of different treatment measures on the remission level of suicidal ideation under multiple time nodes**

**Database search keywords and information**

Search Name:

Cochrane library

ID Search Hits

#1 MeSH descriptor: [Ketamine] explode all trees 2780

#2 MeSH descriptor: [Suicidal Ideation] explode all trees 1048

#3 MeSH descriptor: [Depression] explode all trees 18679

#4 #1 and #2 and #3 31

Embase:

#1 AND #2

115

#2

'ketamine'/exp OR 'ketamine'

65,207

#1

('suicidal ideation'/exp OR 'suicidal ideation') AND ('depression'/exp OR 'depression') AND ('clinical trial'/de OR 'randomized controlled trial'/de)

2,230

115 results

Web of science:

depression(Abstract) and Suicidal ideation(Abstract) and Ktamine(Abstract)

156 results from Web of Science Core Collection

CNKI:

(主题=抑郁) AND (主题=自杀意念)

128 results

PUBMED

Search number Query Sort By Filters Search Details Results

8 (((((((((((((2-(2-Chlorophenyl)-2-(methylamino)cyclohexanone[Title/Abstract]) OR (CI-581[Title/Abstract])) OR (CI 581[Title/Abstract])) OR (CI581[Title/Abstract])) OR (Ketalar[Title/Abstract])) OR (Ketalar[Title/Abstract])) OR (Ketanest[Title/Abstract])) OR (Calipsol[Title/Abstract])) OR (Kalipsol[Title/Abstract])) OR (Calypsol[Title/Abstract])) OR (Ketamine Hydrochloride[Title/Abstract])) OR ("Ketamine"[Mesh])) AND ((((((Depressive Symptoms[Title/Abstract]) OR (Depressive Symptom[Title/Abstract])) OR (Symptom, Depressive[Title/Abstract])) OR (Emotional Depression[Title/Abstract])) OR (Depression, Emotional[Title/Abstract])) OR ("Depression"[Mesh]) "depressive symptoms"[Title/Abstract] OR "depressive symptom"[Title/Abstract] OR "symptom depressive"[Title/Abstract] OR "emotional depression"[Title/Abstract] OR "depression emotional"[Title/Abstract] OR "Depression"[MeSH Terms])) AND ((((Ideation, Suicidal[Title/Abstract]) OR (Ideations, Suicidal[Title/Abstract])) OR (Suicidal Ideations[Title/Abstract])) OR ("Suicidal Ideation"[Mesh]) "ideation suicidal"[Title/Abstract] OR "ideations suicidal"[Title/Abstract] OR "suicidal ideations"[Title/Abstract] OR "Suicidal Ideation"[MeSH Terms]) ((((("2"[All Fields] AND "2-Chlorophenyl"[All Fields]) AND "2"[All Fields]) AND "methylamino"[All Fields]) AND "cyclohexanone"[Title/Abstract]) OR "CI-581"[Title/Abstract] OR "CI-581"[Title/Abstract] OR "CI581"[Title/Abstract] OR "Ketalar"[Title/Abstract] OR "Ketalar"[Title/Abstract] OR "Ketanest"[Title/Abstract] OR "Calipsol"[Title/Abstract] OR "Kalipsol"[Title/Abstract] OR "Calypsol"[Title/Abstract] OR "ketamine hydrochloride"[Title/Abstract] OR "Ketamine"[MeSH Terms]) AND ((("depressive symptoms"[Title/Abstract] OR "depressive symptom"[Title/Abstract] OR "symptom depressive"[Title/Abstract] OR "emotional depression"[Title/Abstract] OR "depression emotional"[Title/Abstract] OR "Depression"[MeSH Terms]) AND "depressive symptoms"[Title/Abstract]) OR "depressive symptom"[Title/Abstract] OR "symptom depressive"[Title/Abstract] OR "emotional depression"[Title/Abstract] OR "depression emotional"[Title/Abstract] OR "Depression"[MeSH Terms]) AND ((("ideation suicidal"[Title/Abstract] OR "ideations suicidal"[Title/Abstract] OR "suicidal ideations"[Title/Abstract] OR "Suicidal Ideation"[MeSH Terms]) AND "ideation suicidal"[Title/Abstract]) OR "ideations suicidal"[Title/Abstract] OR "suicidal ideations"[Title/Abstract] OR "Suicidal Ideation"[MeSH Terms]) 75

7 (((((((((((2-(2-Chlorophenyl)-2-(methylamino)cyclohexanone[Title/Abstract]) OR (CI-581[Title/Abstract])) OR (CI 581[Title/Abstract])) OR (CI581[Title/Abstract])) OR (Ketalar[Title/Abstract])) OR (Ketalar[Title/Abstract])) OR (Ketanest[Title/Abstract])) OR (Calipsol[Title/Abstract])) OR (Kalipsol[Title/Abstract])) OR (Calypsol[Title/Abstract])) OR (Ketamine Hydrochloride[Title/Abstract])) OR ("Ketamine"[Mesh]) (((("2"[All Fields] AND "2-Chlorophenyl"[All Fields]) AND "2"[All Fields]) AND "methylamino"[All Fields]) AND "cyclohexanone"[Title/Abstract]) OR "CI-581"[Title/Abstract] OR "CI-581"[Title/Abstract] OR "CI581"[Title/Abstract] OR "Ketalar"[Title/Abstract] OR "Ketalar"[Title/Abstract] OR "Ketanest"[Title/Abstract] OR "Calipsol"[Title/Abstract] OR "Kalipsol"[Title/Abstract] OR "Calypsol"[Title/Abstract] OR "ketamine hydrochloride"[Title/Abstract] OR "Ketamine"[MeSH Terms] 15,403

6 ((((((((((2-(2-Chlorophenyl)-2-(methylamino)cyclohexanone[Title/Abstract]) OR (CI-581[Title/Abstract])) OR (CI 581[Title/Abstract])) OR (CI581[Title/Abstract])) OR (Ketalar[Title/Abstract])) OR (Ketalar[Title/Abstract])) OR (Ketanest[Title/Abstract])) OR (Calipsol[Title/Abstract])) OR (Kalipsol[Title/Abstract])) OR (Calypsol[Title/Abstract])) OR (Ketamine Hydrochloride[Title/Abstract]) (((("2"[All Fields] AND "2-Chlorophenyl"[All Fields]) AND "2"[All Fields]) AND "methylamino"[All Fields]) AND "cyclohexanone"[Title/Abstract]) OR "CI-581"[Title/Abstract] OR "CI-581"[Title/Abstract] OR "CI581"[Title/Abstract] OR "Ketalar"[Title/Abstract] OR "Ketalar"[Title/Abstract] OR "Ketanest"[Title/Abstract] OR "Calipsol"[Title/Abstract] OR "Kalipsol"[Title/Abstract] OR "Calypsol"[Title/Abstract] OR "ketamine hydrochloride"[Title/Abstract] 1,046

5 "Ketamine"[Mesh] Most Recent "Ketamine"[MeSH Terms] 15,040

4 (((((Depressive Symptoms[Title/Abstract]) OR (Depressive Symptom[Title/Abstract])) OR (Symptom, Depressive[Title/Abstract])) OR (Emotional Depression[Title/Abstract])) OR (Depression, Emotional[Title/Abstract])) OR ("Depression"[Mesh]) "depressive symptoms"[Title/Abstract] OR "depressive symptom"[Title/Abstract] OR "symptom depressive"[Title/Abstract] OR "emotional depression"[Title/Abstract] OR "depression emotional"[Title/Abstract] OR "Depression"[MeSH Terms] (("depressive symptoms"[Title/Abstract] OR "depressive symptom"[Title/Abstract] OR "symptom depressive"[Title/Abstract] OR "emotional depression"[Title/Abstract] OR "depression emotional"[Title/Abstract] OR "Depression"[MeSH Terms]) AND "depressive symptoms"[Title/Abstract]) OR "depressive symptom"[Title/Abstract] OR "symptom depressive"[Title/Abstract] OR "emotional depression"[Title/Abstract] OR "depression emotional"[Title/Abstract] OR "Depression"[MeSH Terms] 188,289

3 (((Ideation, Suicidal[Title/Abstract]) OR (Ideations, Suicidal[Title/Abstract])) OR (Suicidal Ideations[Title/Abstract])) OR ("Suicidal Ideation"[Mesh]) "ideation suicidal"[Title/Abstract] OR "ideations suicidal"[Title/Abstract] OR "suicidal ideations"[Title/Abstract] OR "Suicidal Ideation"[MeSH Terms] (("ideation suicidal"[Title/Abstract] OR "ideations suicidal"[Title/Abstract] OR "suicidal ideations"[Title/Abstract] OR "Suicidal Ideation"[MeSH Terms]) AND "ideation suicidal"[Title/Abstract]) OR "ideations suicidal"[Title/Abstract] OR "suicidal ideations"[Title/Abstract] OR "Suicidal Ideation"[MeSH Terms] 13,422

2 "Suicidal Ideation"[Mesh] "Suicidal Ideation"[MeSH Terms] 13,017

1 "Depression"[Mesh] "Depression"[MeSH Terms] 152,344
